# Mitochondrial Protein UQCRC1 is Oncogenic and a Potential Therapeutic Target for Pancreatic Cancer

**DOI:** 10.7150/thno.38704

**Published:** 2020-01-12

**Authors:** Qing Wang, Mengge Li, Yu Gan, Shuheng Jiang, Jie Qiao, Wei Zhang, Yingchao Fan, Yuling Shen, Yanfang Song, Zihong Meng, Ming Yao, Jianren Gu, Zhigang Zhang, Hong Tu

**Affiliations:** 1 State Key Laboratory of Oncogenes and Related Genes, Shanghai Cancer Institute, Renji Hospital, Shanghai Jiao Tong University School of Medicine, Shanghai, China.; 2 Department of Head and Neck Surgery, Renji Hospital, Shanghai Jiao Tong University School of Medicine, Shanghai, China.

**Keywords:** Pancreatic Ductal Adenocarcinoma, UQCRC1, Mitochondrial Oxidative Phosphorylation, Extracellular ATP

## Abstract

**Purpose**: Pancreatic ductal adenocarcinoma (PDAC) is a malignant disease with a poor prognosis. One prominent aspect of PDAC that contributes to its aggressive behavior is its altered cellular metabolism. The aim of this study was to characterize the oncogenic effects of ubiquinol-cytochrome c reductase core protein I (UQCRC1), a key component of mitochondrial complex III, in PDAC development and to assess its potential as a therapeutic target for PDAC.

**Experimental Design**: The expression of UQCRC1 in human PDAC tissues and p48-Cre/p53Flox/WT/LSL-KrasG12D (KPC) mouse pancreatic intraepithelial neoplasias (PanINs) was determined by immunohistochemistry. The role of UQCRC1 in promoting PDAC growth was evaluated *in vitro* in PANC-1 and CFPAC-1 cells and* in vivo* in transplanted mouse models of PDAC. Extracellular flux and RNA-Seq analyses were applied to investigate the mechanism of UQCRC1 in the regulation of mitochondrial metabolism and PDAC cell growth. The therapeutic potential of UQCRC1 in PDAC was assessed by knockdown of UQCRC1 using an RNA interference approach.

**Results**: UQCRC1 expression showed a gradual increase during the progression from PanIN stages to PDAC in KPC mice. Elevated expression of UQCRC1 was observed in 72.3% of PDAC cases and was correlated with poor prognosis of the disease. UQCRC1 promoted PDAC cell growth in both *in vitro* experiments and *in vivo* subcutaneous and orthotopic mouse models. UQCRC1 overexpression resulted in increased mitochondrial oxidative phosphorylation (OXPHOS) and ATP production. The overproduced ATP was released into the extracellular space via the pannexin 1 channel and then functioned as an autocrine or paracrine agent to promote cell proliferation through the ATP/P2Y2-RTK/AKT axis. UQCRC1 knockdown or ATP release blockage could effectively inhibit PDAC growth.

**Conclusion**: UQCRC1 has a protumor function and may serve as a potential prognostic marker and therapeutic target for PDAC.

## Introduction

Pancreatic ductal adenocarcinoma (PDAC) is one of the most lethal human malignancies whose incidence is increasing. Despite advances in the treatment of PDAC, its 5-year survival rate still remains at ~8% 1. Therefore, there is an urgent need to explore novel approaches to treat PDAC. Reprogramming of cellular metabolism is considered a hallmark of cancer 2. Pancreatic cancer displays extensively reprogrammed metabolism 3, which is driven by oncogene-mediated cell-autonomous pathways 4 or by interactions with noncancer cells in the tumor microenvironment 5. Metabolism therefore stands out as a promising target for the development of novel anti-PDAC agents.

Cancer cells preferentially use glycolysis for ATP production. For decades, tumor cells have been considered defective in mitochondrial respiration due to their dominant glycolytic metabolism 6. However, tumor cells that preferentially rely on aerobic glycolysis for energy production can switch metabolic phenotypes to ATP generation through oxidative phosphorylation (OXPHOS) by interrupting aerobic glycolysis, demonstrating that neoplastic cells maintain the capacity to perform OXPHOS 7. Indeed, increased OXPHOS has been observed in multiple cancer types, exemplifying that OXPHOS can also be utilized in oncogenic metabolism 8, 9. In PDAC, across a panel of 30 pancreatic cancer cell lines, only 13% of cell lines predominantly rely on glycolysis. Most of the other PDAC cell lines utilize OXPHOS for energy generation 10. Enhanced OXPHOS can facilitate PDAC cell growth 11. Moreover, pancreatic cancer stem cells 12 and dormant PDAC cells, which are responsible for tumor relapse 13, rely on oxidative metabolism for survival. Notably, most of our knowledge on the mitochondrial metabolism of PDAC comes from cell or animal experiments, and little is known about its actual status in the context of a clinical setting. There is a lack of investigations focusing on the expression changes of mitochondrion-localized proteins, particularly those that play important roles in energy metabolism, in PDAC.

Targeting mitochondrial metabolism is an attractive strategy for cancer therapy 14, 15. In our previous study, we reported that mice rearing in an enriched environment (EE) with increased space, enhanced social interactions and physical activity displayed a PDAC-resistant phenotype due to eustress stimulation 16, 17. Integrative analysis of transcriptomic and proteomic data revealed that in response to EE, the differentially expressed genes in PDAC cells were largely enriched in the citrate cycle (TCA) and OXPHOS pathways 17. Notably, most of the differentially expressed genes related to mitochondrial respiration were downregulated by EE, supporting that mitochondria may be a major target for PDAC remission induced by EE. Ubiquinol-cytochrome c reductase core protein I (UQCRC1), a key subunit of complex III of the mitochondrial respiratory chain 18, repeatedly showed significantly reduced expression in the PDAC xenograft mouse model under EE conditions. UQCRC1 is homologous to mitochondrial-processing peptidase, which catalyzes the maturity of complex III 19. UQCRC1 plays a critical role in electron transport and ATP generation 20. UQCRC1 deregulation has been reported to be involved in a variety of disorders, such as schizophrenia 21, Rett syndrome 22 and inherited insulin resistance 23. However, despite its increased expression in osteosarcoma cells 24, breast cancer and ovarian tumor 25, little is known about its biological impact on carcinogenesis.

In the present study, we investigated the role of UQCRC1 in PDAC. Our results revealed that UQCRC1 promotes PDAC growth by increasing mitochondrial OXPHOS and ATP production. Targeting UQCRC1 expression and ATP release can effectively suppress PDAC growth. As UQCRC1 expression is shown to be upregulated in approximately 72% of tumor tissues, our study suggests UQCRC1 as a new molecular target for PDAC intervention.

## Materials and Methods

### Cell culture and treatment regents

The human PDAC cell lines AsPC-1, BxPC-3, PANC-1, and CFPAC-1 were obtained from the American Type Culture Collection (ATCC) and cultured according to the culture methods recommended by ATCC. Normal human pancreatic HPNE and HPDE6C7 cells were preserved at Shanghai Cancer Institute and cultured as described before 26. All cell lines were authenticated by Biowing Applied Biotechnology Co., Ltd. (Shanghai, China). The AZD4547 (CSNpharm, Arlington Heights, IL, USA), INC280 (CSNpharm) and AR-C118925 (R&D, Minneapolis, MN, USA) inhibitors were reconstituted in DMSO (Sigma-Aldrich, St. Louis, MO, USA) and used at final concentrations of 1 μM, 1 μM and 5 μM, respectively. Reactive blue 2 (RB2, Sigma-Aldrich), 10Panx (APExBIO, Shanghai, China), iso-PPADS (Tocris, St. Louis, MO, USA), metformin (APExBIO), ATP standard (Promega, Madison, WI, USA) were reconstituted in phosphate-buffered saline (PBS) and used at final concentrations of 50 μM, 100 μM, 100 μM, 10 mM and 10 nM, respectively.

### Tissue microarrays and immunohistochemistry

This study was conducted in accordance with the International Ethical Guidelines for Health-related Research Involving Humans and was approved by the Research Ethics Committee of Renji Hospital. A total of 159 pathologically confirmed PDACs and 141 adjacent normal pancreatic tissues were collected by Shanghai National Engineering Research Center from Taizhou Hospital from 2010 to 2014. The antibodies used for immunohistochemistry (IHC) targeted UQCRC1 (Proteintech, Rosemont, IL, USA), PANX1 (Proteintech), PCNA (Proteintech) and cytokeratin 19 (CK-19, Proteintech). The intensity of the staining was evaluated as follows: negative, 0 points; weak, 1 point; moderate, 2 points; strongly positive, 3 points. The percentage of positive tumor cells was scored as 0 (< 5%), 1 (< 25%), 2 (25%-50%), 3 (51%-75%), and 4 (> 75%). Scores (1 to 12) were based on the percent of positive cells and staining intensity within the tissues.

### Public database

Information on PDAC in TCGA was used for the Cox regression analysis and Gene Set Enrichment Analysis (GSEA). We classified the PDAC patients into two groups according to the median mRNA expression level of UQCRC1: patients with UQCRC1 expression above the median value were classified as the high expression group, while patients with UQCRC1 expression below the median value were classified as the low expression group. GSEA was performed using the software described previously 27. Comparison of *UQCRC1* expression in PDAC patients from the TCGA with that in the normal Genotype-Tissue Expression (GTEx) database was performed by Gene Expression Profiling Interactive Analysis (GEPIA).

### Constructions of stable transgenic cell lines

Full-length cDNA encoding human *UQCRC1* was amplified by PCR and cloned into the pCDH-CMV-MCS lentiviral vector (Lv) system. Primers for UQCRC1 overexpression construction were UQCRC1-F: 5'-CCGCTAGCGCCACCATGGCGGCGTCCGTGGTCTGTC; and UQCRC1-R: 5'-GGGTCGACCTAGAAGCGCAGCCAGAACATGCCG. Sequences of short hairpin RNAs (shRNAs) for UQCRC1 knockdown and PANX1 knockdown were shUQCRC1-1: CATGATGTTCGTCCTGCAA; shUQCRC1-2: ACAAGCTATGCCAGAGTT; and shPANX1-1: GGTCACATGTATTGCCGT. Plasmids for lentiviral packaging were transfected into 293T cells with Lipofectamine 2000 (Invitrogen, Carlsbad, CA, USA). PANC-1 and CFPAC-1 cells grown at 60%-70% confluence were infected with the viral particle supernatant. Stable UQCRC1 knockdown or overexpressing cell clones were obtained by limiting dilution and verified by qPCR and Western blotting.

### RNA-Seq

Briefly, total RNA from ATP-treated (16 h), UQCRC1-overexpressing and control PANC-1 cells was isolated using TRIzol reagent according to the manufacturer's instructions (ThermoFisher, Waltham, MA, USA). After construction, cDNA library sequencing was performed using an Illumina, Hiseq X10 platform by BGI Genetic Corporation (Wuhan, China). High-quality reads were aligned to the human reference genome (GRCh38) using Bowtie2. Gene expression was calculated from fragments per kilobase of transcript per million (FPKM) by expectation maximization (RSEM). The transcript profiles of this study were submitted to the BioSample Submission Portal as Bio-Project PRJNA513941, and Sequence Read Archive (SRA) accession numbers were ranked from SRR8422342 to SRR8422350. Gene ontology (GO) term and KEGG pathway enrichment of our RNA-Seq profiles was performed by GSEA as described above.

### Quantitative real-time PCR

Total RNA was isolated as described above, and cDNA was synthesized using 2 μg of total RNA with PrimeScript™ RT Master Mix (Takara, Kusatsu, Shiga, Japan). Quantitative real-time PCR (qPCR) was subsequently carried out with the FastStart Universal SYBR Green Master (Rox) qPCR (Roche, Indianapolis, IN, Switzerland) kit. *ACTB* was utilized as an internal control. Relative expression levels of genes were determined by the ΔΔCt method. The qPCR primers used in this study are listed in Table S1.

### Cell proliferation assay

The effect of UQCRC1 on the cell proliferation of PANC-1 and CFPAC-1 was evaluated by real-time cell analysis (RTCA) with an E-plate 16 (ACEA Biosciences, San Diego, CA, USA). For statistical analysis, the cell index (CI) values were normalized at the point of cell seeding. Cell function in response to treatment was assessed with the CellTiter 96 CCK8 assays (Dojindo, Kumamoto, Japan) at 48 h according to the manufacturer's instructions, and the optical density (OD) was measured at 450 nm. Each experiment contained three replicates per condition and was repeated three times.

### Colony formation assay

Briefly, cells were trypsinized and resuspended to generate a single-cell suspension and seeded into 6 cm dishes in triplicate. After 2-3 weeks of incubation, the colonies were fixed with 4% paraformaldehyde and then stained with 1% crystal violet. The number of colonies was counted with ImageJ software.

### Bromodeoxyuridine incorporation assay

Cells were incubated with 10 μM bromodeoxyuridine (BrdU) solution (Abcam, Cambridge, MA, USA) for 16 h at 37 °C and then permeabilized with 0.3% Triton X-100 for 10 min. After washing three times, cells were incubated with Alexa Fluor 647 anti-BrdU antibody (BioLegend, San Jose, CA, USA) for 30 min at room temperature. Data were acquired using a flow cytometer with an excitation of 630 nm.

### Cell cycle analysis

Cells were collected and fixed in 1 mL of 70% methanol in PBS for 1 h at 4 °C. The cells were resuspended in 1 mL of 50 μg/mL propidium iodide (PI, Abcam) solution for half an hour and then subjected to flow cytometric analysis with a FACScan flow cytometer.

### Measurement of intracellular ATP and extracellular ATP

The intracellular and extracellular ATP concentrations were quantified using the rLuciferase/Luciferin reagent (Promega) according to the manufacturer's instructions. Briefly, 1×10^6^ cells were lysed with 0.5% trichloroacetic acid (pH 7.75) at 4 °C for 30 min, and 100 μL cell lysate or cultural supernatant was mixed with 100 μL rLuciferase/Luciferin reagent for 3 s. The intensity of the emitted light was measured using a GloMax 20/20 illuminometer. The ATP concentration was determined by plotting an ATP standard curve and was normalized to cell number.

### Measurement of intracellular NAD^+^/NADH ratio

The oxidized and reduced forms of intracellular nicotinamide adenine dinucleotide (NAD) were determined using the NADH/NAD^+^ Quantification Kit (Beyotime, Shanghai, China) according to the manufacturer's instructions. Briefly, 1×10^6^ cells were lysed with 200 μL of NADH/NAD^+^ extraction buffer. The extracted NADH/NAD^+^ supernatant (100 μL) was heated to 60 °C for 30 min to obtain a pure NADH supernatant by decomposing NAD^+^. A volume of 20 μL of NADH/NAD^+^ or NADH supernatant was mixed with 90 μL of alcohol dehydrogenase and incubated at 37 °C for 10 min to convert NAD^+^ to NADH. The absorbance was measured at 450 nm after adding 10 μL of developing solution per well. The NAD^+^/NADH ratio was calculated according to the protocol.

### Measurement of intracellular ADP/ATP ratio

The ADP/ATP ratio was assayed using an ADP/ATP Ratio Assay Kit (Abcam). Briefly, cell samples treated with nucleotide releasing buffer were mixed with ATP-monitoring enzyme for ATP measurement. The above mixture was then incubated with ADP-converting enzyme to measure ADP levels. The ADP/ATP ratio was calculated according to the protocol.

### Extracellular flux analysis

The oxygen consumption rate (OCR) was measured using an XFe96 extracellular analyzer (Agilent, Santa Clara, CA, USA) according to the manufacturer's instructions. Briefly, after being starved in serum-free medium for 16 h, 1.5×10^4^ cells were cultured in 80 μL of growth medium at 37 °C in 5% CO_2_ for 12-24 h. Before being washed with 180 μL of assay medium, cells were preincubated at 37 °C without CO_2_ for 1 h. OCR was measured under basal conditions before the sequential addition of 20 μL of oligomycin (1 μM), 22 μL of carbonyl cyanide 4-(trifluoromethoxy) phenylhydrazone (FCCP, 0.5 μM), and 25 μL of rotenone/antimycin A (1 μM) to the indicated final concentrations in each well. The values were calculated after normalization to the total cell protein and are plotted as the mean ± SEM.

### Animal experiments

To construct a subcutaneous PDAC model, UQCRC1-overexpressing or knockdown PANC-1 cells (4×10^6^) were injected into the right flanks of five-week-old male BALB/c nude mice. For the pharmacological study of subcutaneous xenografts, animals in each independent experiment were randomized into two groups one week after the inoculation of tumor cells. For the 10Panx treatment (n = 6), 10Panx was dissolved in PBS to a final concentration of 100 μM and injected intratumorally every day. The control group (n = 6) was injected with an equivalent volume of PBS. For the metformin treatment (n = 6), 0.50 g metformin was dissolved in 1 L of water to reach a final oral administration concentration of 100 mg/kg/d per mouse, and the control group (n = 6) was provided with water. Tumor volume was calculated using the equation volume = 0.50 × length × width^2^. For the orthotopic xenograft study, UQCRC1-overexpressing or knockdown PANC-1 cells (2×10^6^) were mixed with Matrigel (Corning, Corning, NY, USA) and inoculated into the head/neck of mouse pancreas. PDAC transgenic p48-Cre/p53Flox/WT/LSL-KrasG12D (KPC) mice were generated as described previously 28. The animal studies were approved by the Animal Care and Use Committees of Renji Hospital.

### Statistical analysis

Data were analyzed with GraphPad Prism 6 and are shown as the mean ± SEM. Student's t-test or ANOVA were applied to compare statistically significant differences (*P* < 0.05) between two or more groups. Independent samples from different groups that did not conform to a normal distribution were analyzed with the Wilcoxon signed-rank test. Comparison of Kaplan-Meier survival curves was performed with the log-rank Mantel-Cox test. The independent effect of UQCRC1 expression on overall survival was evaluated using multivariate Cox regression models in SAS software.

### Other Experimental Assays

Other supporting verifications and functional assays are described in the Supplementary Materials and Methods.

## Results

### UQCRC1 is upregulated in PDAC and correlates with disease prognosis

**Figure 1:**
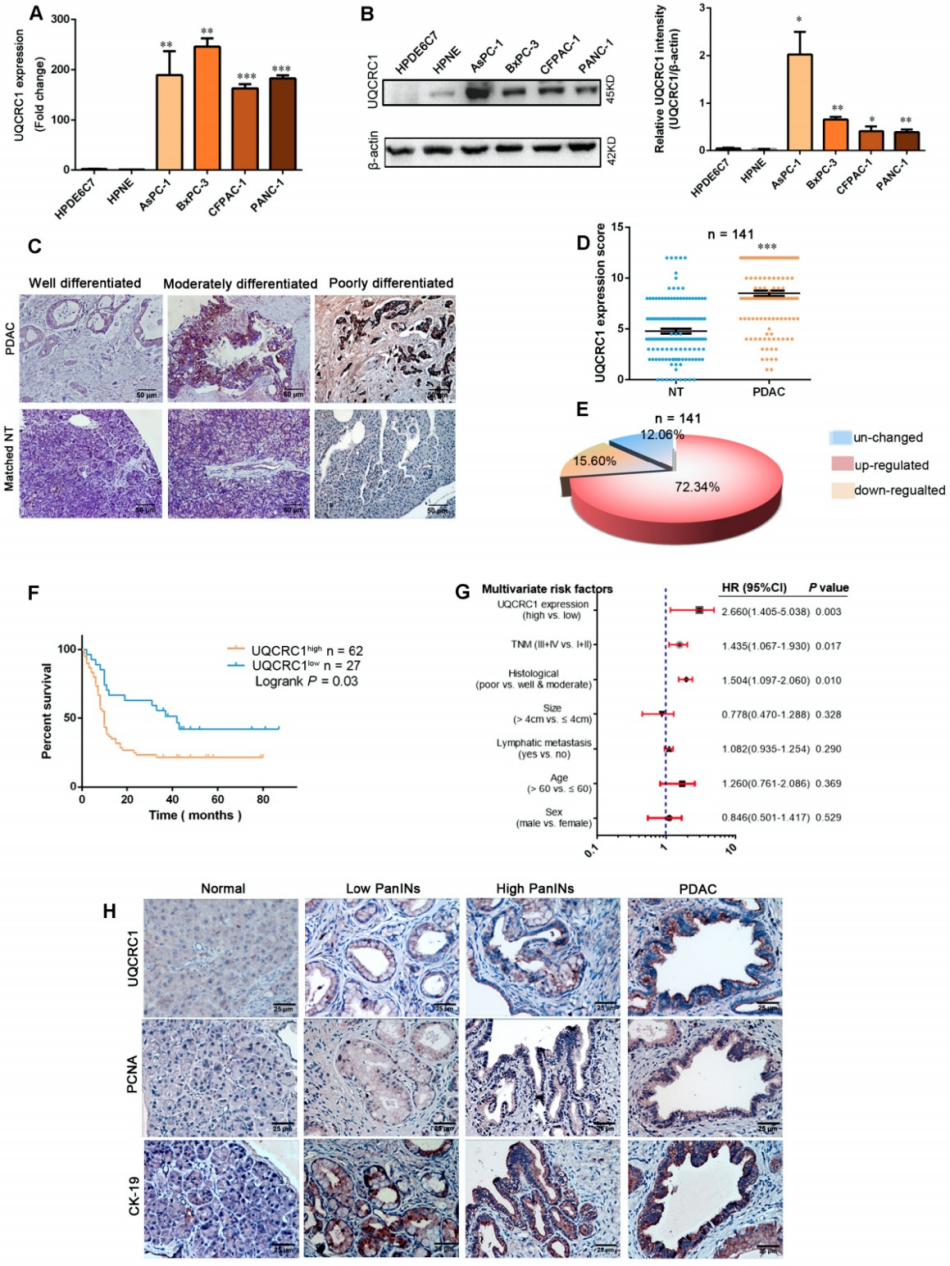
** UQCRC1 is upregulated in PDAC and correlates with disease prognosis. (A)** Relative mRNA levels of *UQCRC1* in PDAC cells and normal pancreatic cells (HPDE6C7 and HPNE) as determined by qPCR. **(B)** Protein levels of UQCRC1 in PDAC cells and normal pancreatic cells as determined by Western blotting (n = 3). **(C)** Representative IHC images of UQCRC1 in PDAC tissues with different differentiation statuses and in adjacent nontumor pancreatic tissues (NT, nontumor tissues). **(D)** IHC scores of UQCRC1 staining in PDAC tissues and matched adjacent nontumor tissues (*P* < 0.001, n = 141, NT, nontumor tissues). **(E)** The percentage of PDAC cases with normal or aberrant expression of UQCRC1 (n = 141). **(F)** Kaplan-Meier analysis of OS based on the UQCRC1 levels in PDAC (n = 89). **(G)** Multivariate Cox regression analysis of the data from the TMA cohort revealed that UQCRC1 was an independent predictor of OS (n = 89). **(H)** Representative IHC images of UQCRC1, PCNA and CK-19 in PanINs and PDAC tissues from KPC mice. *P < 0.05; **P < 0.01; ***P < 0.001.

We first investigated the expression of UQCRC1 in a panel of human PDAC cell lines, namely, AsPC-1, BxPC-3, CFPAC-1 and PANC-1. UQCRC1 expression was higher at both the mRNA (**Figure 1A**) and protein levels (**Figure 1B**) in the 4 PDAC cell lines than it was in the two normal pancreatic ductal cells HPDE6C7 and HPNE. Then, we compared the mRNA expression level of *UQCRC1* in 179 PDAC and 171 nontumor pancreatic tissues using data from the TCGA and GTEx databases. Upregulation of *UQCRC1* was also observed in PDAC cases (*P* < 0.05, **Figure S1A**). We further examined UQCRC1 expression in two TMAs containing a total of 159 PDAC and 141 paracancer specimens. Enhanced IHC staining of UQCRC1 was observed in the cytoplasm of PDAC cells, particularly in poorly differentiated PDAC cases (**Figure 1C**). In 141 paired samples, 102 had increased expression of UQCRC1 in the PDAC specimens (*P* < 0.0001, **Figure 1D-E**). Clinicopathological association analysis with pooled data from TCGA and TMA cohorts revealed that increased UQCRC1 was positively correlated with tumor size, histological grade and TNM stage of human PDAC (**Table 1**).

We also analyzed the correlation between UQCRC1 expression and clinical outcomes in 89 cases from the TMA cohort and 90 cases from TCGA that had prognostic information. Compared with the patients with low UQCRC1 in tumors, those with high UQCRC1 demonstrated worse overall survival (OS) (TMA cohort, 5-year median OS: 10.0 *vs*. 42.0 months, *P* < 0.01, **Figure 1F**) and a higher chance for postsurgical relapse (TCGA cohort, 5-year median disease free survival: 18.3 *vs.* 55.6 months, *P* < 0.05, **Figure S1B**). Multivariate Cox regression analyses showed that UQCRC1 expression was an independent predictor of PDAC aggressiveness, with the most significant hazard ratios (HR) for predicting clinical prognosis in both TMA (**Figure 1G**) and TCGA (**Figure S1C**) cohorts.

To understand the UQCRC1 status in precancerous lesions, we enrolled a genetically engineered KPC mouse model of PDAC. In wild-type animals, the basal levels of UQCRC1 in normal pancreatic ducts and acini were very low. However, UQCRC1 expression showed a gradual increase from PanIN1 to PanIN3, though to a lesser extent than PDAC (**Figure 1H, 1^st^ row**). This IHC staining pattern was similar to the cell proliferative marker PCNA was similar (**Figure 1H, 2^nd^ row**). The epithelial phenotype of PanINs was confirmed by staining with the epithelial cell marker CK-19 (**Figure 1H, 3^rd^ row**). These results indicated that UQCRC1 upregulation occurs early, before PDAC formation.

### UQCRC1 promotes PDAC growth *in vitro* and *in vivo*

**Figure 2:**
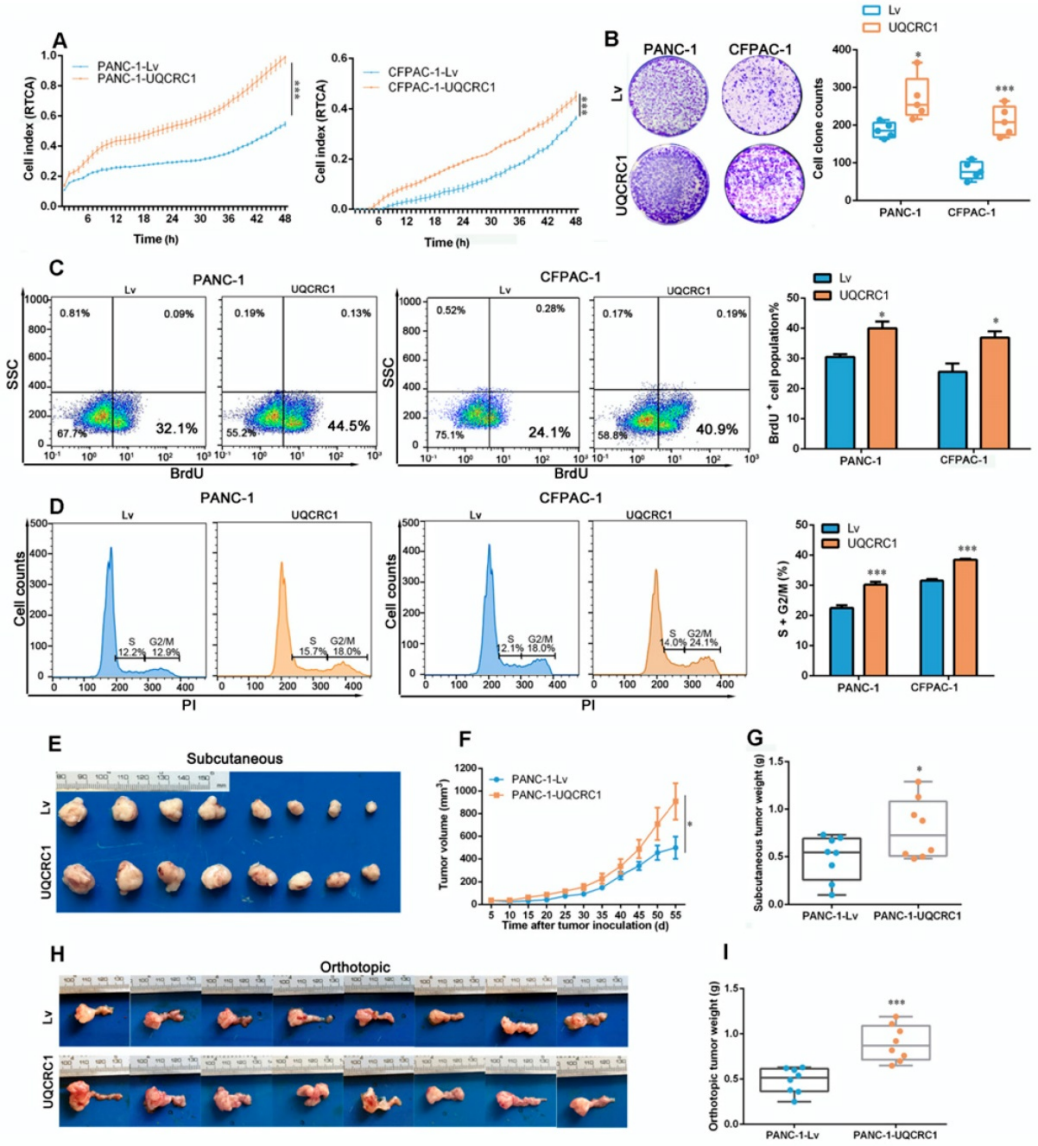
** UQCRC1 promotes PDAC cell growth *in vivo* and *in vitro.* (A)** Cell growth was increased in UQCRC1-overexpressing PANC-1 and CFPAC-1 cells as measured with RTCA (n = 4, two-way ANOVA). **(B)** Representative images and quantitative analysis of the enhanced colony formation ability of UQCRC1-overexpressing PDAC cells (n = 5). **(C)** Cell proliferation was increased in UQCRC1-overexpressing PANC-1 and CFPAC-1 cells as indicated by BrdU incorporation. **(D)** Cell proportions in the S and G2/M phases of the cell cycle were increased in UQCRC1-overexpressing PANC-1 and CFPAC-1 cells compared to control cells. **(E-G)** The subcutaneous xenografts, tumor growth curves and the tumor weights in nude mice inoculated with UQCRC1-overexpressing PANC-1 cells and the control cells for 56 days (n = 8, *P* < 0.05, two-way ANOVA for tumor volume and t-test for tumor weight). **(H-I)** Orthotopic xenografts and tumor weights of nude mice inoculated with UQCRC1-overexpressing PANC-1 cells and control cells after 56 days (n = 8, *P* < 0.001, two-way ANOVA or tumor volume and t-test for tumor weight). *P < 0.05; **P < 0.01; ***P < 0.001.

To investigate the functional role of UQCRC1 in PDAC, we overexpressed UQCRC1 in PDAC cell lines by using lentiviral vectors. Because UQCRC1 was highly expressed in all four PDAC cell lines that we examined (**Figure 1A-B**), we chose two cell lines, namely, PANC-1 and CFPAC-1, which had relatively low intrinsic expression of UQCRC1 to perform the functional study. Analyses of qPCR and Western blotting confirmed the successful overexpression of UQCRC1 in PANC-1 and CFPAC-1 cells (**Figure S2A-B**). Confocal microscopy revealed that ectopic expression of UQCRC1 was mainly located in the mitochondrial compartment (**Figure S2C**). The results from the RTCA (**Figure 2A**) and colony formation assay (**Figure 2B**) revealed that UQCRC1 overexpression significantly increased the cell growth rates of PANC-1 and CFPAC-1 cells. The BrdU incorporation assay indicated that UQCRC1 promoted cell proliferation (*P* < 0.05, **Figure 2C**). This was also supported by the cell cycle analysis showing increased cell populations of S and G2/M phases (**Figure 2D**). UQCRC1 overexpression had no influence on cell apoptosis (**Figure S2D**). Apart from cell proliferation and apoptosis, we also measured the migration and invasion capabilities of UQCRC1-overexpressing PANC-1 and CFPAC-1 cells but found no changes compared to control cells (**Figure S2E-G**). Collectively, these data indicated that UQCRC1 mainly plays a positive role in regulating PDAC cell growth.

To further confirm the role of UQCRC1 in PDAC progression, we conducted in *vivo* experiments using subcutaneous and orthotopic mouse models of PDAC. Consistent with our *in vitro* findings, UQCRC1 overexpression significantly increased the tumor burden in both subcutaneous (0.49 ± 0.08 g *vs.* 0.79 ± 0.11 g,* P* < 0.05, **Figure 2E-G**) and orthotopic (0.48 ± 0.05 g *vs.* 0.90± 0.07 g,* P* < 0.001, **Figure 2H-I**) mouse models of PDAC.

### Overexpression of UQCRC1 enhances mitochondrial OXPHOS and ATP generation

**Figure 3:**
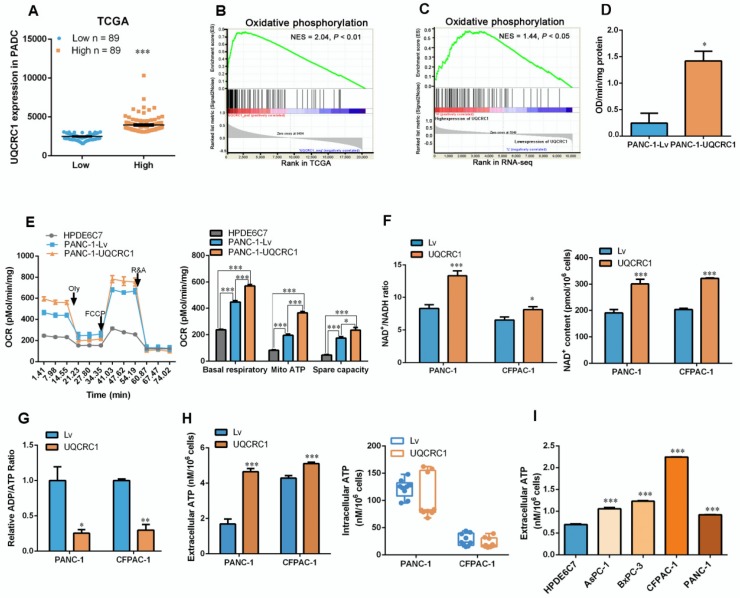
** Overexpression of UQCRC1 enhances mitochondrial oxidative phosphorylation and ATP generation in PDAC cells. (A)** Classification of PDAC patients from the TCGA database according to the mRNA expression level of UQCRC1. **(B)** Upregulation of UQCRC1 expression was positively correlated with activated OXPHOS metabolic pathways in PDAC as predicted by GSEA (n = 179, GSEA, gene set enrichment analysis; NES, normalized enrichment score, *P* < 0.01). **(C)** UQCRC1 overexpression was associated with OXPHOS pathway enrichment by GSEA of RNA-Seq data (FPKM, fragments per kilobase of transcript per million > 1, a total of 10113 genes) from PANC-1-UQCRC1 and control cells. **(D)** Activity of mitochondrial complex III was enhanced in PANC-1-UQCRC1 compared with control cells. **(E)** Increased OCRs in UQCRC1-overexpressing PANC-1 cells as measured by the XFe96 extracellular analyzer (n = 3; Oly, oligomycin, 1 μM; FCCP, carbonyl cyanide 4-(trifluoromethoxy) phenylhydrazone, 1 μM; R&A, rotenone and antimycin A, 1 μM; Mito, mitochondria). **(F)** NAD+/NADH ratio and NAD+ content in UQCRC1-overexpressing PANC-1 and CFPAC-1 cells and the control cells as determined by using the NADH/NAD+ Quantification Kit. **(G)** ADP/ATP ratio in UQCRC1-overexpressing PANC-1 and CFPAC-1 and the control cells as determined by using the ADP/ATP Ratio Assay Kit. **(H)** ATP content in the cell culture medium and the intracellular compartment of UQCRC1-overexpressing PANC-1 and CFPAC-1 cells as detected by rLuciferase/Luciferin reagents. **(I)** ATP content in the cell culture medium of 4 PDAC cell lines and one normal pancreatic cell line (HPDE6C7). *P < 0.05; **P < 0.01; ***P < 0.001.

GSEA of the TCGA datasets was used to search the molecular pathways that were significantly associated with *UQCRC1* expression. PDAC patients were classified into two groups according to the mRNA expression level of* UQCRC1* (**Figure 3A**). GSEA results showed that *UQCRC1* was mostly associated with genes involved in OXPHOS (NES = 2.04, *P* < 0.01, **Figure 3B**). We then performed GSEA using our own RNA-Seq data (FPKM > 1, a total of 10113 genes) generated from PANC-1-Lv and PANC-1-UQCRC1 cells and found that the genes altered by UQCRC1 overexpression were significantly associated with OXPHOS (**Figure 3C**, *P* < 0.05), PI3K-AKT signaling, and E2F targets (**Figure S3A**, *P* < 0.05). In addition, we also performed GO analysis with GSEA and found that the differentially expressed genes were mostly enriched in ATPase regulator activity, protein localization to chromosome and chaperone binding (**Figure S3B**, *P* < 0.01). These results confirmed our TCGA findings that UQCRC1 overexpression promoted OXPHOS flux in PDAC cells. Furthermore, the genes encoding mitochondrial complexes (e.g., *NDUFS6*, *NDUFB4*, *UQCRFS1*, *SDHA*, *ATP5L2*) and OXPHOS regulators (e.g., *SIRT1*, *SIRT2*, *TFAM*, *PPARGC1A*) were significantly upregulated in the UQCRC1-overexpressing cells compared to the control cells (Adjusted *P* < 0.05,**Figure S3C**); Of these genes, *SIRT1*, *SIRT2*, *PPARGA1A*, *TFAM*, *NDUFS6*, *UQCRFS1* and *ATP5L2* were confirmed to be significantly upregulated in PANC-1-UQCRC1 cells by qPCR analysis **(Figure S3D)**, suggesting that the increased expression of UQCRC1 may affect mitochondrial energy metabolism.

To verify whether UQCRC1 overexpression affects OXPHOS, we first examined the activity of mitochondrial complex III and found that UQCRC1 overexpression accelerated the catalytic rate of complex III (**Figure 3D**). The mitochondrial respiration capacity in UQCRC1-overexpressing cells was then determined by detecting the OCR after treatment with different mitochondrial complex inhibitors. We found that the basal OCRs in UQCRC1-overexpressing PANC-1 and CFPAC-1 cells were both higher than those in their corresponding control cells (*P* < 0.001, **Figure 3E, Figure S3E**). After the addition of oligomycin, a mitochondrial complex V inhibitor, OCRs fell below the basal level. The reduced OCR levels in UQCRC1-overexpressing cells were more evident than those in control cells (*P* < 0.001, **Figure 3E, Figure S3E**), indicating that overexpression of UQCRC1 enhanced mitochondria-dependent respiration. Instead, FCCP, a mitochondrial uncoupler, stimulated higher maximal respiration in UQCRC1-overexpressing PDAC cells, implying that the uncoupled maximal respiratory capacity for ATP synthesis was also enhanced by UQCRC1 overexpression. In normal pancreatic HPDE6C7 cells, the basal, mitochondrion-dependent and maximum respirations were all lower than those in two PDAC cell lines. As OXPHOS is important for supporting NAD^+^ recycling, we then measured the content of NAD^+^ and the ratio of NAD^+^/NADH in PDAC cells with or without UQCRC1 overexpression. The results showed that the ratio of NAD^+^/NADH in UQCRC1-overexpressing cells was significantly increased due to the increase in NAD^+^ content (**Figure 3F, Figure S3F**), indicating that the rate of electron transport in the respiratory chain was increased with UQCRC1 overexpression. Moreover, the decrease in the ADP/ATP ratio of UQCRC1-overexpressing cells further supported the enhancement of OXPHOS by UQCRC1 overexpression (**Figure 3G**).

Since the ultimate function of OXPHOS is to generate ATP, we measured the intracellular and extracellular ATP (eATP) content and found that UQCRC1 overexpression increased ATP concentrations in cell culture supernatants but not in cell lysates (**Figure 3H**). We then compared the eATP levels of 4 PDAC cell lines with the normal pancreatic cell line HPDE6C7. As expected, ATP concentrations in the culture medium from each of the four PDAC cell lines were higher than those in the medium of nonmalignant HPDE6C7 cells (**Figure 3I**). These results indicated that ATP generated through enhanced mitochondrial OXPHOS in PDAC cells was largely released into the cell culture medium rather than being accumulated inside cells.

### eATP is released from PANX1 channels and mediates UQCRC1-induced cell proliferation

**Figure 4:**
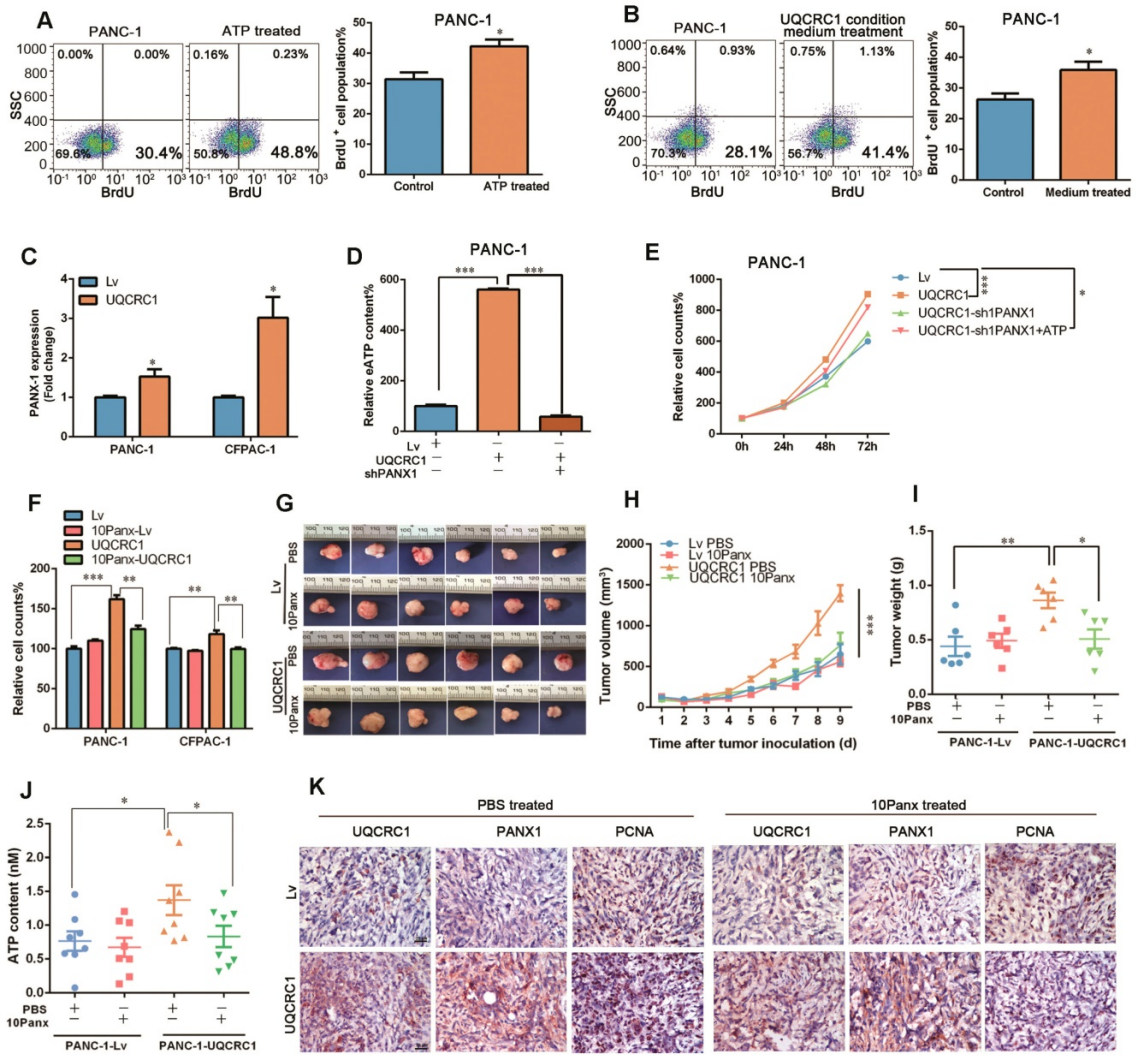
** eATP is released from the PANX1 channel and mediates UQCRC1-induced cell proliferation. (A)** Treatment with 10 nM ATP for 16 h enhanced the proliferation of PANC-1 cells as measured by BrdU incorporation. **(B)** Treatment with conditioned medium from UQCRC1-overexpressing PANC-1 cells for 16 h enhanced the proliferation of PANC-1 cells. **(C)** Relative mRNA expression levels of the *PANX1* gene in UQCRC1-overexpressing PANC-1 and CFPAC-1 cells. **(D)** ATP content was decreased in the cell culture medium of PANC-1-UQCRC1 cells with the knockdown of PANX1 (*P* < 0.001). **(E)** The increased cell growth rate of PANC-1 induced by UQCRC1 overexpression was attenuated by PANX1 knockdown, and this attenuation could be largely reversed by 10 nM ATP supplementation for 16 h (n = 4, two-way ANOVA). **(F)** Cell growth of PDAC cells enhanced by UQCRC1 overexpression was attenuated after treatment with 100 μM 10Panx for 48 h, as detected by the CCK8 assay (n = 4). **(G-I)** The subcutaneous xenografts, tumor growth curves and tumor weights in nude mice inoculated with UQCRC1-overexpressing PANC-1 cells and control cells treated with PBS or 100 μM 10Panx intratumorally every day (n = 6, two-way ANOVA for tumor volume and t-test for tumor weight). **(J)** Serum ATP content in tumor-bearing mice after treatment with PBS or 10Panx (n = 6). **(K)** Representative IHC images of UQCRC1, PANX1 and the proliferation marker PCNA in subcutaneous xenografts under the indicated treatment conditions (n = 6, scale bar, 25 μm). *P < 0.05; **P < 0.01; ***P < 0.001.

As the ATP concentration in the cell culture medium was significantly increased with the overexpression of UQCRC1, we asked whether soluble ATP could serve as a signal molecule to promote PDAC cell proliferation. Exogenous ATP stimulation promoted the growth of PANC-1 cells in a dosage-dependent manner (**Figure S4A**). The BrdU incorporation assay confirmed that ATP supplementation could promote the proliferation of PANC-1 cells (**Figure 4A**). Notably, conditioned medium from UQCRC1-overexpressing PANC-1 cells accelerated the proliferation of control cells by 13.7% (**Figure 4B**), indicating that eATP released from PANC-1-UQCRC1 cells played a role, at least partly, in medicating UQCRC1-induced cell growth.

To date, five ATP-permeable channels, i.e., connexin hemichannels and pannexin 1 (PANX1), calcium homeostasis modulator 1, volume-regulated anion and maxi-anion channels, have been documented to mediate ATP release. Among these channels, only PANX1 was found to be upregulated in UQCRC1-overexpressing cells based on our RNA-Seq results (**Figure S4B,** RNA-Seq profiles from SRR8422342 to SRR8422350). The qPCR results confirmed the increase of PANX1 in UQCRC1-overexpressing PANC-1 and CFPAC-1 cells (**Figure 4C, Figure S4C**). To assess the biological impact of PANX1 on ATP release, we knocked down the *PANX1* gene in UQCRC1-overexpressing PANC-1 cells by a shRNA approach. Western blotting results indicated that *PANX1* was successfully knocked down (**Figure S4D**). The increased ATP concentration in the cell culture medium of PANC-1-UQCRC1 cells was largely reduced due to accumulation in the cytoplasm after *PANX1* gene knockdown (*P* < 0.001, **Figure 4D, Figure S4E**), indicating that the PANX1 channel was indispensable for ATP release in UQCRC1-overexpressing cells. Moreover, blocking ATP release by *PANX1* knockdown attenuated the cell growth promoted by UQCRC1 overexpression, and this decreased promotion could be recovered by additional ATP stimulation (**Figure 4E**). Similar to the results of RNA interference, 10Panx, a specific chemical inhibitor of PANX1 29, was also able to abolish UQCRC1-induced PDAC cell growth enhancement (**Figure 4F**). Consistent with our *in vitro* findings, in mice receiving 10Panx intratumor injections, the protumor effect of UQCRC1 was largely attenuated (tumor weight of the PANC-1-UQCRC1-PBS group 0.86 ± 0.07 g *vs.* tumor weight of the PANC-1-UQCRC1-10Panx group 0.51 ± 0.09 g, *P* < 0.05), while the tumor growth was not affected by 10Panx in the PANC-1-Lv-PBS group and the PANC-1-Lv-10Panx group (0.44 ± 0.09 g *vs.* 0.49 ± 0.06 g, *P* > 0.05, **Figure 4G-I**). The serum ATP concentrations were markedly reduced upon treatment with 10Panx in mice bearing UQCRC1-overexpressing tumors (**Figure 4J**). IHC of UQCRC1, PANX1 and PCNA confirmed that blocking ATP release with 10Panx could reduce cell proliferation in UQCRC1-overexpressing xenografts (**Figure 4K**). Taken together, these data indicated that ATP was released via the PANX1 channel and that this channel played an important role in UQCRC1-induced cell proliferation.

### UQCRC1 promotes PDAC cell proliferation through the eATP/P2Y2-RTK/AKT axis

**Figure 5:**
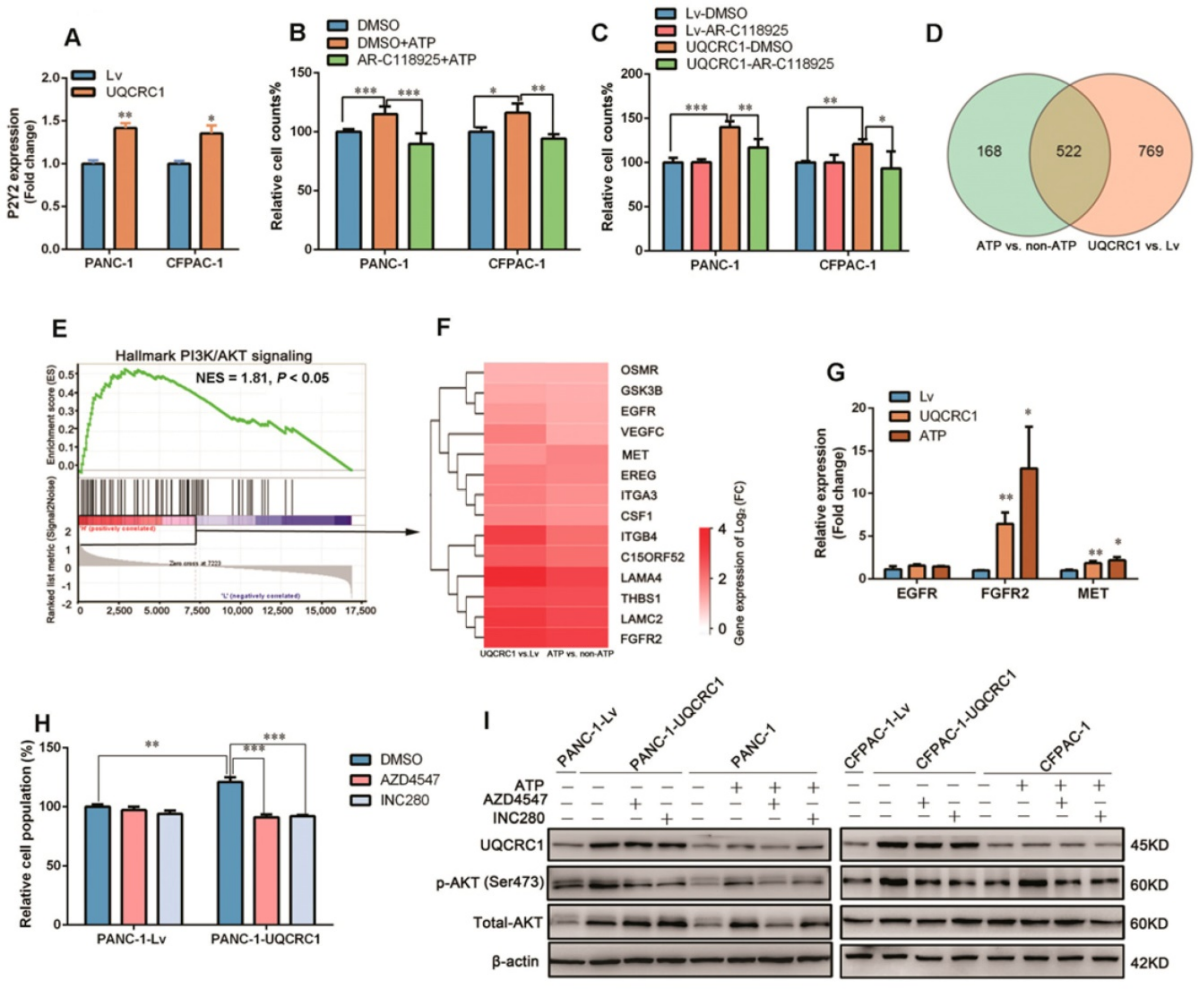
** UQCRC1 promotes PDAC growth through the eATP/P2Y2-RTK/AKT axis. (A)** Relative mRNA levels of *P2Y2* in UQCRC1-overexpressing PANC-1 and CFPAC-1 cells (n = 3). **(B-C)** Relative cell growth enhanced by 10 nM ATP stimulation for 48 h or UQCRC1 overexpression in PANC-1 and CFPAC-1 cells was abolished after treatment with 5 μM AR-C118925 for 48 h, as detected by the CCK8 assay (n = 4). **(D)** Overlap of differentially expressed genes from ATP-treated PANC-1 cells and PANC-1-UQCRC1 cells compared with control cells based on the RNA-Seq profiles. **(E)** GSEA analysis of the 522 changed genes showed that the PI3K/AKT signaling pathway was activated in both UQCRC1-overexpressing and ATP-treated PANC-1 cells. **(F)** The heat map of enriched PI3K/AKT-related genes, according to log2 (FC) ≥ 0.5 and FPKM ≥ 1 (n = 3, FC, fold change).** (G)** Relative mRNA levels of *EGFR*, *FGFR*2 and *MET* genes in UQCRC1-overexpressing PANC-1 cells (n = 3). **(H)** Relative cell growth enhancement by UQCRC1 overexpression in PANC-1 was abolished after treatment with 1 μM FGFR2 inhibitor AZD4547 or 1 μM MET inhibitor INC280 for 48 h (n = 4). **(I)** Western blot of UQCRC1, p-AKT, and total AKT in UQCRC1-overexpressing and control PANC-1 and CFPAC-1 cells treated with 10 nM ATP, ATP plus 1 μM AZD4547 or ATP plus 1 μM INC280 for 16 h. *P < 0.05; **P < 0.01; ***P < 0.001.

To characterize which subtype of adenosine receptors triggers eATP signaling in PDAC, we treated cell lines with two antagonists (iso-PPADS and RB2) against P2X and P2Y, respectively, in PANC-1 cells. The cell proliferation enhancement caused by ATP stimulation or UQCRC1 overexpression was inhibited by the P2Y inhibitor RB2 but not by the P2X inhibitor iso-PPADS (**Figure S5A-C**). Of the two well-defined P2Y subtypes P2Y2 and P2Y11, only *P2Y2* was shown to be upregulated in UQCRC1-overexpressing cells (**Figure 5A, Figure S5D**). Moreover, AR-C118925, an antagonist specific for P2Y2, fully blocked the cell proliferative effect induced by ATP supplementation or UQCRC1 overexpression (**Figure 5B-C**). These results indicated that P2Y2 was the functional receptor for eATP in PANC-1 and CFPAC-1 cells.

To explore the signaling pathway downstream of the P2Y2 receptor, we performed RNA-Seq and found that a total of 522 genes had expression changes with both ATP stimulation and UQCRC1 overexpression (**Figure 5D**). GSEA analysis of these 522 genes revealed that they were mostly enriched in the PI3K-AKT signaling pathway (NES = 1.81, *P* < 0.05, **Figure 5E**). As the G protein-coupled receptor P2Y2 usually cross talks with receptor tyrosine kinases (RTKs) to exert its function 28, we then examined the expressions of three RTKs, i.e., *EGRF*, *FGFR2* and *MET*, which were identified by RNA-Seq to be affected by treatment with ATP and forced expression of UQCRC1 (**Figure 5F**). The qPCR results indicated that *FGFR2* and *MET* were significantly upregulated in PANC-1 cells after ATP treatment or UQCRC1 overexpression (**Figure 5G**). To further confirm the role of FGFR2 and MET in the eATP-triggered signaling pathway, we treated PANC-1-UQCRC1 and PANC-1-Lv cells with AZD4547, a selective FGFR2 receptor antagonist, and INC280, a specific c-MET inhibitor. It was demonstrated that the enhancement of cell proliferation induced by UQCRC1 overexpression was significantly attenuated by either AZD4547 or FGFR2 (*P* < 0.001,** Figure 5H**), suggesting that both of these two RTKs participated in eATP signal transduction.

To assess the involvement of AKT in eATP-induced cell proliferation, we performed a Western blotting assay to determine AKT and phosphorylated AKT (p-AKT) levels in PDAC cells. Consistent with the findings from RNA-Seq, ATP treatment or UQCRC1 overexpression caused an increased expression of total AKT in PANC-1 cells (**Figure 5I**). Meanwhile, p-AKT levels in ATP-treated or UQCRC1-overexpressing PANC-1 and CFPAC-1 cells were significantly higher than those in control cells (**Figure 5I**), suggesting that the AKT signaling pathway was activated by ATP stimulation or UQCRC1 overexpression. Moreover, both the FGFR2 inhibitor AZD4547 and the MET inhibitor INC280 could greatly diminish AKT phosphorylation levels induced by ATP treatment or UQCRC1 overexpression, further supporting that AKT is the downstream signaling molecule of eATP/P2Y2-RTK. Taken together, these data suggest that UQCRC1 promotes PDAC cell proliferation through the eATP/P2Y2-RTK/AKT axis.

### Knocking down UQCRC1 inhibits the growth of PDAC and overexpressing UQCRC1 sensitizes PDAC to metformin therapy

**Figure 6:**
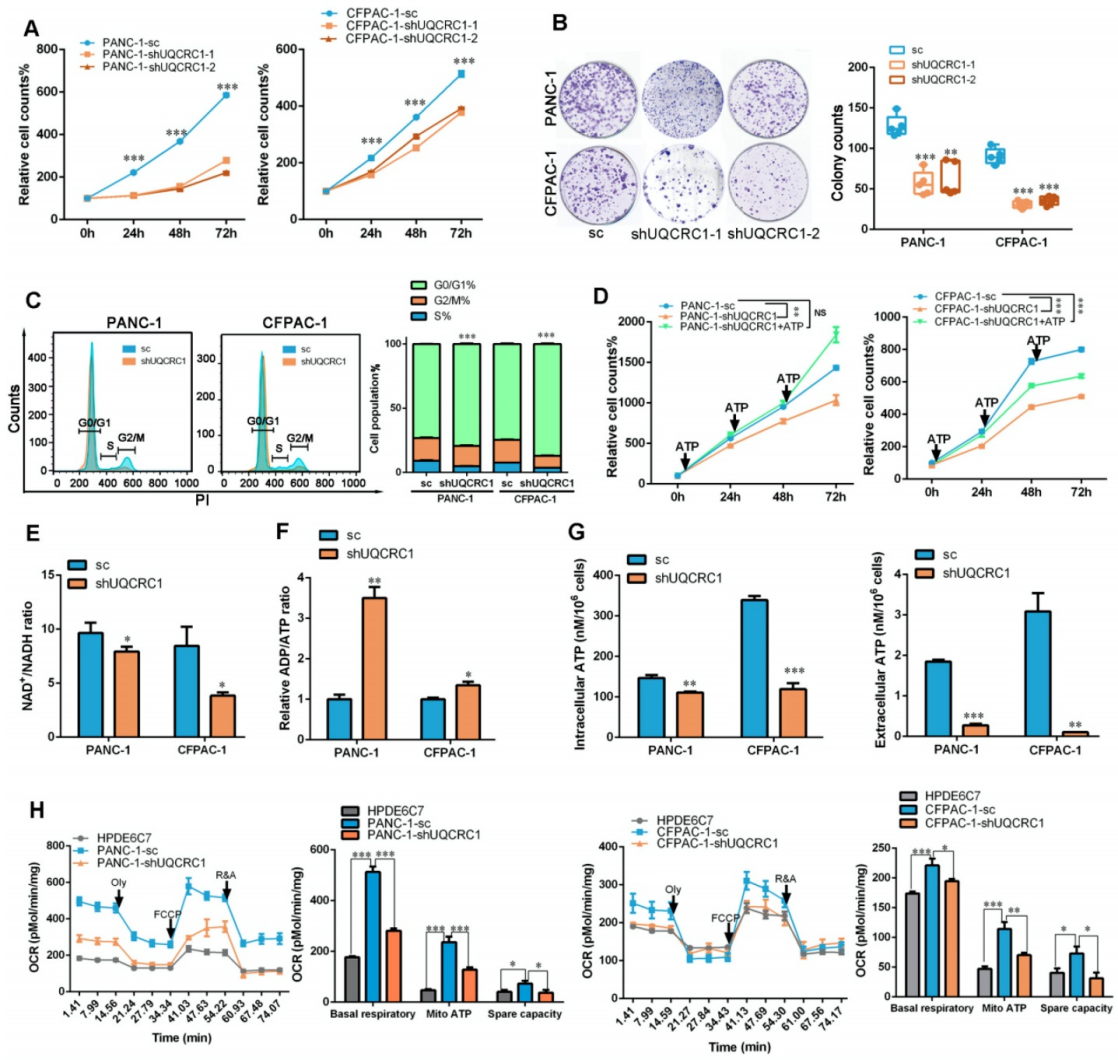
** UQCRC1 knockdown suppresses PDAC cell growth by inhibiting mitochondrial OXPHOS and ATP generation. (A)** Relative cell growth in UQCRC1 knockdown PANC-1 and CFPAC-1 cells as measured by CCK8 (n = 4, sc: scramble, two-way ANOVA). **(B)** Representative images and quantitative analysis of the declined colony formation ability of UQCRC1 knockdown PANC-1 and CFPAC-1 cells (n = 5). **(C)** UQCRC1 knockdown caused G0/G1 phase accumulation (t-test for G0/G1 phase and *P* < 0.001). **(D)** Relative cell growth of PANC-1 and CFPAC-1 cells stably expressing shUQCRC1 in the presence or absence of 10 nM ATP for 48 h (n = 4, two-way ANOVA). **(E)** Ratio of NAD^+^/NADH in PANC-1 and CFPAC-1 cells with or without UQCRC1 downregulation (n = 4). **(F)** Ratio of ADP/ATP in PANC-1 and CFPAC-1 cells with or without UQCRC1 downregulation (n = 4). **(G)** Intracellular ATP and eATP content of PANC-1 and CFPAC-1 cells with or without UQCRC1 downregulation. **(H)** Decreased OCRs in PANC-1 and CFPAC-1 cells with UQCRC1 downregulation (n = 3). *P < 0.05; **P < 0.01; ***P < 0.001.

To investigate whether UQCRC1 may serve as a potential target for PDAC therapy, we knocked down UQCRC1 by using an RNA interference approach in PANC-1 and CFPAC-1 cells, which exhibited relatively higher endogenous expression levels of UQCRC1 than the normal pancreatic cell lines HPDE6C7 and HPNE (**Figure 1A-B and Figure S6A-B**). Knocking down UQCRC1 led to a significantly decreased rate of PDAC cell growth, as shown in the CCK8 and colony formation assays. Moreover, cell cycle analysis revealed that knocking down UQCRC1 caused G0/G1 phase accumulation (**Figure 6A-C**). This cell growth inhibition effect could be reversed by ATP supplementation (**Figure 6D**), suggesting that eATP and its related cell growth signals were diminished after UQCRC1 knockdown. The decreased NAD^+^/NADH ratio (**Figure 6E**), increased ADP/ATP ratio (**Figure 6F**), and reduced contents of intracellular and eATP (**Figure 6G**) in PANC-1-shUQCRC1 cells consistently supported that UQCRC1 knockdown impeded mitochondrial OXPHOS and ATP generation. Extracellular flux analysis demonstrated that RNA interference against *UQCRC1* inhibited mitochondria-dependent respiration (**Figure 6H**). Flow cytometry analysis showed that UQCRC1 knockdown did not cause apparent apoptosis (**Figure S6C**)**.** The results of transmission electron microscopy revealed an increased number of autolysosomes in PANC-1-shUQCRC1 cells (**Figure S6D-E**), suggesting that energy deficiency induced autophagy to sustain energy homeostasis and cell survival.

**Figure 7:**
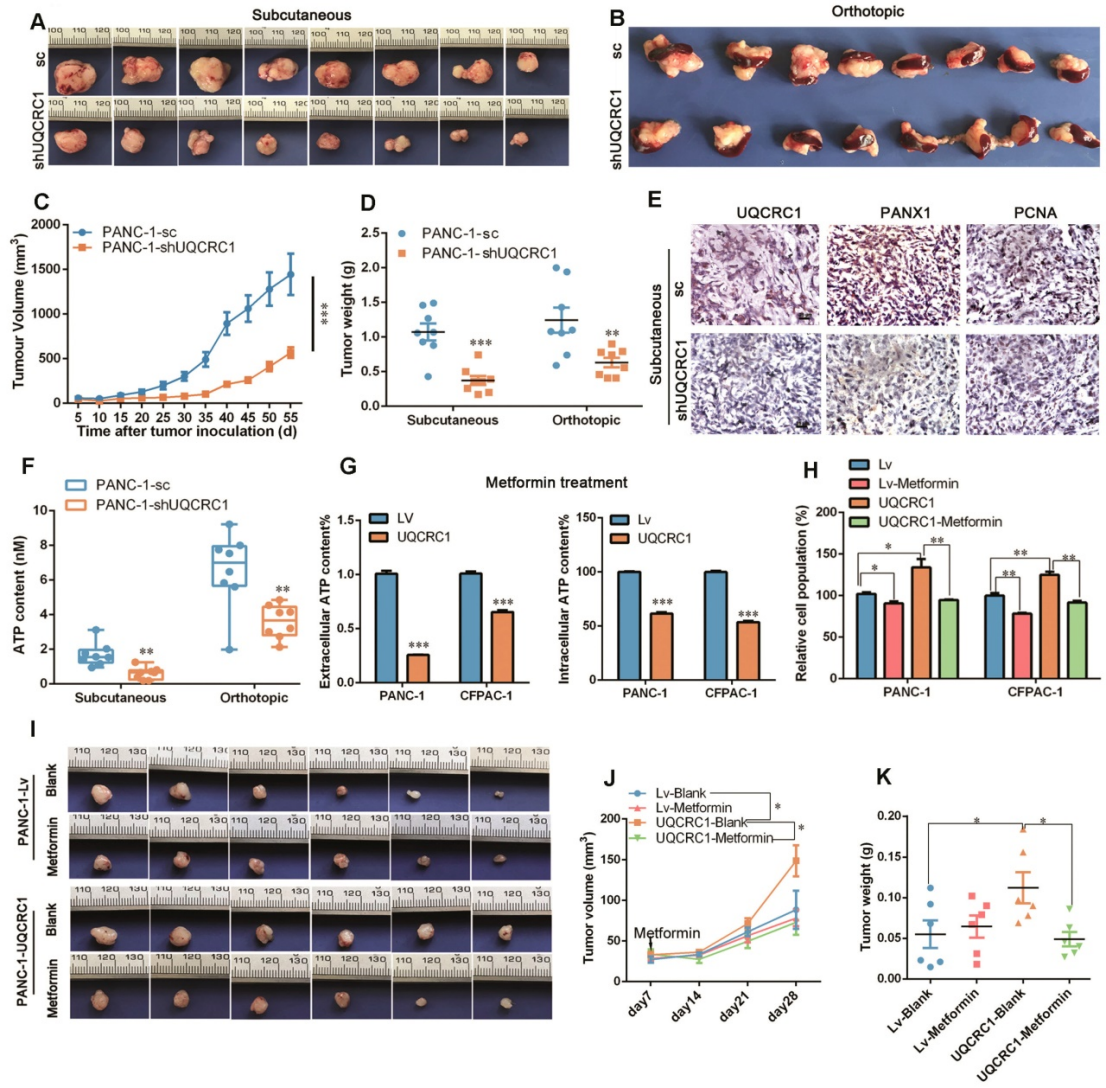
** Targeting UQCRC1 and mitochondrial OXPHOS effectively inhibits PDAC growth. (A-B)** Photographs of the subcutaneous and orthotopic xenograft tumors formed by UQCRC1 knockdown and control PANC-1 cells (n = 8). **(C-D)** Tumor growth curves and the tumor weights of subcutaneous and orthotopic xenografts at 56 days after tumor cell inoculation (n = 8, two-way ANOVA for tumor volume and t-test for tumor weight). **(E)** Representative IHC images of UQCRC1, PANX1 and the proliferation marker PCNA in subcutaneous xenografts generated from PANC-1-sc and PANC-1-shUQCRC1 cells (n = 8, scale bar, 25 μm). **(F)** Serum ATP content in tumor-bearing mice subcutaneously or orthotopically inoculated with PANC-1-sc and PANC-1-shUQCRC1 cells (n = 8). **(G)** Relative eATP (left panel) and intracellular ATP content (right panel) of PANC-1 and CFPAC-1 cells treated with or without 10 mM metformin for 48 h. **(H)** Relative cell growth enhanced by UQCRC1 overexpression in PANC-1 and CFPAC-1 cells was abolished after treatment with 10 mM metformin for 48 h as determined by the CCK8 assay. **(I-K)** The subcutaneous xenografts, tumor growth curves and tumor weights of nude mice, which were fed with 100 mg/kg/day metformin or water for three weeks after inoculation with UQCRC1-overexpressing PANC-1 cells or control cells (n = 6, *P* < 0.05, two-way ANOVA for tumor volume and t-test for tumor weight). *P < 0.05; **P < 0.01; ***P < 0.001.

In the *in vivo* experiments, both subcutaneous and orthotopic mouse models demonstrated that the tumors formed by UQCRC1 knockdown PANC-1 cells grew at a much slower rate than those formed by the control cells (1.07 ± 0.12 g *vs.* 0.37 ± 0.07 g, *P* < 0.001 in the subcutaneous mouse model; 1.24 g ± 0.18 g *vs.* 0.63 ± 0.07 g, *P* < 0.01 in the orthotopic model, **Figure 7A-D**). PCNA immunohistochemistry demonstrated that xenograft tumors formed by PANC-1-shUQCRC1 cells had a reduced cell proliferation rate (**Figure 7E**). The IHC results also indicated that PANX1 expression was downregulated in PANC-1-shUQCRC1 cells, suggesting that the release of ATP may be impaired in PANC-1-shUQCRC1 cells due to the reduced number of PANX1 channel (**Figure 7E**). Consistent with this observation, ATP concentrations in the blood of mice transplanted with PANC-1-shUQCRC1 cells were significantly lower than those in control mice (**Figure 7F**).

As enhanced mitochondrial OXPHOS provides ATP to sustain PDAC proliferation, we used metformin, an electron transfer chain complex I inhibitor, to investigate the differential impacts of OXPHOS-targeted therapy on UQCRC1-overexpressing and control PDAC cells. As shown in Figure 7G, metformin treatment at 10 mM decreased eATP and intracellular ATP content in PANC-1 and CFPAC-1 cells (*P* < 0.001, **Figure 7G**). As expected, treatment with 10 mM metformin inhibited PANC-1 and CFPAC-1 cell growth by 11.5% (*P* < 0.05) and 21.8% (*P* < 0.01), respectively. Strikingly, when UQCRC1 was overexpressed in PANC-1 and CFPAC-1 cells, the cell growth inhibition rates in response to metformin reached 39.3% (*P* < 0.01) and 33.4% (*P* < 0.01), respectively (**Figure 7H**). In the PDAC subcutaneous model, metformin at the concentration of 100 mg/kg/day did not show an inhibitory effect on tumors formed by PANC-1-Lv cells (0.06 ± 0.02 g *vs.* 0.06 ± 0.01 g, *P* > 0.05), but it effectively inhibited the growth of tumors formed by UQCRC1-overexpressing cells (0.11 ± 0.02 g *vs.* 0.05 ± 0.01 g, *P* < 0.05 **Figure 7I-K**). Collectively, these results suggest that metformin was more effective in cancers whose growth was dependent on mitochondrial OXPHOS.

## Discussion

In the current study, we investigated, for the first time, the biological impact of the mitochondrial protein UQCRC1 in cancer. We found that UQCRC1 was upregulated in human PDAC cell lines and tissues. This conclusion is supported not only by our own TMA results but also by data from the public cancer databases. In addition, taking advantage of the KPC mice, we were able to demonstrate that the increased expression of UQCRC1 occurs in PanIN stages, although to a lesser extent than in fully transformed PDAC. Previously, dysregulation of UQCRC1 was reported to be associated with several disorders, including metabolic diseases 23, neuropsychic diseases 30, and reproductive system diseases 31. In human cancers, UQCRC1 expression is upregulated in osteosarcoma 24, colorectal cancer 32, breast cancer and ovarian cancer 25, but downregulated in gastric cancer 33 and clear cell renal cell carcinoma 34. This discrepancy may be due to the different energy metabolic patterns in distinct types of cancers. For instance, it was reported that renal cell carcinoma is characterized by OXPHOS deficiency, which forces the renal tumor cells to perform aerobic glycolysis to produce ATP 35. In contrast, oxidative metabolism prevails over glycolytic metabolism for ATP supply in fast-growing tumors such as breast cancer, ovarian cancer and lung cancer 36. In our study, the increased expression of UQCRC1 was demonstrated in 72.3% of PDAC samples. This phenomenon implies that mitochondrial respiratory function is activated in most PDAC cases. In addition, the increased expression of UQCRC1 in PanIN stages also supports that the growth of abnormal pancreatic ductal cells largely relies on mitochondrial respiration for ATP supply. Although early studies suggested that PDAC is a highly glycolytic tumor in which upregulated GLUT1 increases intracellular glucose levels and promote glycolysis 37, accumulating evidence has revealed that mitochondrial OXPHOS synergistically contributes to ATP generation in PDAC cells 11, 38. Recently, Zhou et al. reported that HSP60, a mitochondria-localized quality control protein, was upregulated in PDAC and promoted cancer cell proliferation via maintaining OXPHOS function to generate ATP 11. Moreover, lipogenic PDAC cell lines 39 and cancer stem cells 12, 13 display a strong dependence on mitochondrial OXPHOS. Here, we provide clinical evidence to support that mitochondrial OXPHOS is important for PDAC progression. It is worth pointing out that our conclusion does not exclude the role of the glycolysis pathway in PDAC. Indeed, we even found increases in glucose uptake and hexokinase II (HK2) activity in UQCRC1-overexpressing PDAC cells (data not shown). On the other hand, we found that in MIAPaCa-2 cell line, which was characterized as a glycolysis-dominant PDAC line 10, UQCRC1 expression level was comparable to that in the OXPHOS-dependent cell lines such as AsPC-1, BxPC-3, CFPAC-1 and PANC-1(data not shown). These findings implied that both glycolysis and OXPHOS contribute to the energy supply for PDAC.

To our knowledge, the oncogenic properties of UQCRC1 have not been previously reported. Our results from both *in vitro* and* in vivo* studies, including those using orthotopic PDAC mouse models, clearly demonstrated that UQCRC1 promotes PDAC growth. How UQCRC1, a mitochondrial complex protein, exerts its protumor effect is an enigma. From the RNA-Seq profiles and qPCR analysis, we noted that a number of mitochondrial complex genes, such as *SIRT1, SIRT2, PPARGC1A, NDUFS6*, *UQCRFS1* and* ATP5L2,* were upregulated by the ectopic expression of UQCRC1. In contrast, UQCRC1 downregulation significantly decreased the expression of these genes (data not shown). The upregulation of multiple OXPHOS-related genes provides possibilities for mitochondrial respiratory enhancement. Because both the absolute amount of NAD^+^ and the ratio of NAD^+^/NADH are increased in UQCRC1-overexpressing PDAC cells, we speculate that the metabolic intermediate NAD^+^ may play a role in coordinating the transcription of mitochondrion-related genes. It is well documented that SIRT1 deacetylase is activated by NAD^+^ 40. Once activated, SIRT1 modifies the function of PGC-1a 41, a transcription coactivator that can regulate the expression of genes related to OXPHOS 42. Interestingly, in our study, we found that overexpression of UQCRC1 resulted in a significantly higher expression of *SIRT1* and *PPARGC1A* (*PGC-1α*). Therefore, this assumption may at least partially explain how increased UQCRC1 influences the expression of other mitochondrial complex genes to achieve synergistic promotion of the OXPHOS program.

A growing number of studies have shown that ATP is not only a molecule for the storage of energy but is also a potent signaling agent in the extracellular environment 43. ATP levels in the tumor interstitial space can be 103 to 104 times higher than those in normal tissues of the same cell origin 44. ATP promotes cancer growth and metastasis through autocrine or paracrine signaling loops, affecting not only cancer cells but also immune cells 45. Because overexpression of UQCRC1 caused a significant increase in ATP in the cell culture medium rather than in the cell lysates in our study, we focused our research on the impact of extracellular ATP on PDAC. We proposed an ATP/P2Y2-RTK/AKT axis through which UQCRC1 promotes PDAC growth. This is in line with other studies showing that blocking purinergic receptors P2Y12 or P2Y2 with pharmaceutical antagonists inhibits PDAC and prolongs orthotopic PDAC mice survival 28, 46. Apart from the purinergic receptor antagonists, this study suggests two other strategies for PDAC drug design, i.e., interfering with the expression or function of mitochondrial complex proteins such as UQCRC1 or disrupting ATP-releasing channels such as PANX1. Whether concomitant intervention of multiple targets can achieve stronger inhibition effects on PDAC growth is an interesting issue that warrants further investigation.

Another novel finding of this study is that PDAC cells overexpressing UQCRC1 are more vulnerable to metformin therapy. Metformin, a biguanide widely prescribed to treat type II diabetes, has been proven to prevent the progression of multiple cancers due to its autonomous inhibition of mitochondrial complex I and cellular respiration 47, 48. Meta-analysis revealed that metformin administration is associated with reduced risk and favorable survival of pancreatic cancer 49, 50. However, some studies, including one recent phase 2 clinical trial 51, did not find any beneficial effect of metformin on PDAC. One explanation for this ineffectiveness is that in some studies, the blood metformin concentrations (7 μmol/L, similar to those for diabetes treatment) may be too low to attenuate the progression of PDAC. PDAC patients with high plasma concentrations of metformin (> 1 mg/L) seemed to have an improved survival rate 51. Interestingly, in this study, we found that at the low concentration of 100 mg/kg/day 52, metformin was effective only in mice bearing tumors overexpressing UQCRC1. This result implied that PDAC patients with high expression of UQCRC1 may benefit more from metformin treatment than those with normal or low levels of UQCRC1. Since PDAC is a highly heterogeneous disease with different metabolic subtypes, including the Warburg phenotype and the reverse Warburg phenotype 3, UQCRC1 may serve as a promising molecular marker for predicting the drug response of PDAC to metformin adjuvant therapy.

The limitation of this study is that we did not explore the upstream pathway that regulates UQCRC1 gene expression in PDAC cells. In addition, this study focused only on the cancer cell-intrinsic mechanism through which eATP promotes PDAC growth but did not investigate the cell-extrinsic mechanisms, particularly the effects of eATP on local cancer immunity. Because ATP and adenosine in the tissue microenvironment can selectively increase the chemotaxis of immunosuppressive Treg cells and facilitate escape of the nascent tumor from immunosurveillance 53, it is worth investigating in the future whether this increased Treg infiltration also occurs in UQCRC1-overexpressing tumors.

Collectively, we report that the upregulation of UQCRC1 is frequently observed in human PDAC and is correlated with the poor prognosis of the patients with disease. This strengthens the importance of OXPHOS metabolism in PDAC and suggests that UQCRC1 can be used as a novel prognostic marker for PDAC. Forced expression of UQCRC1 results in increased mitochondrial respiration, followed by enhanced ATP generation and release. Extracellular ATP triggers the AKT signaling pathway via binding to the P2Y2 receptor and exchanging signals with the membrane RTKs (FGFR2 or c-Met). Targeting UQCRC1 or PANX1 has therapeutic potential against PDAC. Given that UQCRC1 expression is already increased in PanIN stages, our study also opens up a new perspective in the prevention of PDAC by long-term use of metformin in high-risk populations.

## Supplementary Material

Supplementary materials and methods, figures, and table.

## Figures and Tables

**Table 1:** Pooled analysis of the correlation between UQCRC1 expression and clinicopathologic features with PDAC cases from TMA and TCGA cohorts

Clinicopathologic features	Total (n = 320)	UQCRC1		*P* value
		Low (n = 128)	High (n = 192)	
**Sex**				0.3789
Male	187	71	116	
Female	133	57	76	
**Age**				0.3145
<63	169	72	97	
≥63	151	56	95	
**Location**				0.8738
Head	241	97	144	
Body/tail	79	31	48	
**Size (cm)**				0.0176
<4	159	74	85	
≥4	161	54	107	
**Histological grade**				0.0306*
G1	38	20	18	
G2	187	77	110	
G3	95	31	64	
**TNM**				< 0.0001*
I II	153 153	76 45	77 108	
III-IV	14	7	7	
**Lymphatic metastasis**				0.0858
Yes	181	81	100	
No	139	50	89	

Notes: TCGA: The Cancer Genome Atlas; TMA: tissue microarray; TNM: tumor, lymph node, metastasis. * was analyzed with Wilcoxon signed rank test.

## Abbreviations

PDAC: Pancreatic ductal adenocarcinoma
OXPHOS: oxidative phosphorylation
UQCRC1: ubiquinol-cytochrome c reductase core protein I
EE: enriched environment
TCA: the citrate cycle
ATCC: American Type Culture Collection
IHC: immunohistochemistry
TCGA: The Cancer Genome Atlas
GSEA: Gene set enrichment analysis
GTEx: Genotype-Tissue Expression
GEPIA: Gene Expression Profiling Interactive Analysis
Lv: lentiviral vector
FPKM: fragments per kilobase of transcript per million
SRA: sequence read archive
qPCR: quantitative real-time PCR
RTCA: real-time cell analysis
CI: cell index
BrdU: bromodeoxyuridine
PI: propidium iodide
NAD: nicotinamide adenine dinucleotide
OCR: oxygen consumption rate
Oly: oligomycin
FCCP: carbonyl cyanide 4-(trifluoromethoxy) phenylhydrazone
R&A: rotenone/antimycin A
KPC: p48-Cre/p53Flox/WT/LSL-KrasG12D
OS: overall survival
HR: hazard ratios
TMA: tissue microarray
eATP: extracellular ATP
PANX1: pannexin1
TEM: transmission electron microscopy
HK2: hexokinase II
shRNA: short hairpin RNA
NES: normalized enrichment score
PanINs: pancreatic intraepithelial neoplasias
