# Supplementary material for: Mitochondrial Protein UQCRC1 is Oncogenic and a Potential Therapeutic Target for Pancreatic Cancer
Source: Theranostics. 2020 Jan 12;10(5):2141–57. doi: 10.7150/thno.38704 (PMC7019160; doi:10.7150/thno.38704)
Supplement: Supplementary file 1 — Supplementary materials and methods, figures, and table. [file thnov10p2141s1.pdf]

Supplementary Materials and Methods

Cell apoptosis assay

Cells were plated in 6-well plates, and the positive control was induced with 250 μg/mL 5-FU. The apoptosis ratio was analyzed using the Annexin V-PE/7-AAD Apoptosis Detection Kit (BD Biosciences, San Jose, CA, USA) according to the manufacturer's instructions. Briefly, more than 1×10^6 cells were incubated with saturating concentrations of Annexin V-PE and 7-AAD for 20 min at room temperature and immediately analyzed by flow cytometry. Annexin V^+ and 7-AAD^- cells were designated as being in early apoptosis, and Annexin V^+ and 7-AAD^+ cells were designated as signs of late apoptosis. Tests were repeated in triplicate.

Cell migration and invasion assay

Cell migration or invasion was measured using Transwell inserts with an 8 μm pore size culture membrane (Corning, Corning, NY, USA) covered with or without 6.35 mg/mL Matrigel (Corning) before use. Cells were resuspended at 5×10^6 cells/mL in serum-free medium, and 200 μL of cell suspension was added to the upper compartment and incubated at 37 °C, with 700 μL of medium containing 10% FBS in the lower chamber. Cells were stained and counted after being cultured for 8-16 h.

Immunofluorescence staining

The localization of the overexpressed UQCRC1 was detected by confocal laser scanning microscopy. Briefly, tumor cells were stained with MitoTracker Red (Invitrogen, Carlsbad, CA, USA) for 30 min or UQCRC1 antibody (Proteintech, Rosemont, IL, USA) for 1 h and then inoculated with Alexa Fluor 488 Affinipure goat anti-rabbit IgG antibody (Jackson, West Grove,

PA, USA). Cell nuclei were stained with DAPI (ThermoFisher, Waltham, MA, USA) before observation by a fluorescence microscope.

Transmission electron microscopy (TEM)

Cells were harvested and immediately fixed by immersion in 0.1 M phosphate buffer (pH 7.4) containing 2% glutaraldehyde and 2.0% osmium tetroxide. After subsequent washes with phosphate buffer and dehydration through a graded ethanol series, each sample was infiltrated and embedded in epoxy resin. Samples were cut into thin sections, which were mounted on filmed copper grids and stained with uranyl acetate and lead citrate. Grids were examined on a transmission electron microscope.

Western blotting analysis

The protein concentrations were measured with the Pierce BCA Protein Assay Kit (ThermoFisher). Whole-cell protein lysates were separated by SDS-PAGE and then transferred to a PVDF membrane. The membrane was blocked with 5% milk and then incubated with one of the following primary antibodies: anti-UQCRC1 (Proteintech), anti-Akt (Phospho-Ser473) (Signalway Antibody, College Park, Maryland, USA), anti-Akt (Signalway Antibody) or anti-PANX1 (Signalway antibody). Equal protein sample loading was confirmed using an anti-β-actin antibody (Sigma-Aldrich, St. Louis, MO, USA) antibody.

Detection of complex III activity

Briefly, the activity of mitochondrial complex III was determined in a reaction mixture containing 25 mM KH_2PO_4 pH 7.4, 0.05% Tween-20, 4 μM rotenone, and 250 μM fresh decylubiquinone solution. A total of 100 μg isolated mitochondria were coincubated with 250 μM cyt c in the reaction buffer in the presence or absence of 2.5 mM complex III specific inhibitor

antimycin A at 37 °C. Mitochondrial complex III specific activity was calculated by the linear increase of the cyt cox absorbance at 550 nm in a kinetic spectrophotometer. The final data were normalized by protein amount to express the activity as OD/min/mg protein.

Table S1. Primers used in this study

Primers name	Sequences
UQCRC1-F	5'-TGTCTCGTGCAGACTTGACC
UQCRC1-R	5'-GGCGAGGTCTAACAGTTGCT
P2RY2-F	5'-CCCCGTGCTCTACTTTGTCA
P2RY2-R	5'-AGCATGACTGAGCTGTAGGC
P2RY11-F	5'-ACTCAACCACCAGTATGGGC
P2RY11-R	5'-CTGAGGATCGGCACGGGAG
PANX1-F	5'-TCCAAGTTCTTTCTCCTGGCG
PANX1-R	5'-GGGGAAAAACTTATGCAGCCAC
MET-F	5'-TTACGGACCCAATCATGAGC
MET-R	5'-ACTTCGCTGAATTGACCCAT
EGFR-F	5'-AGGAGAACTGCCAGAAACTGACC
EGFR-R	5'-GCCTGCAGCACACTGGTTG
FGFR2-F	5'-TTCGGGGTGTTAATGTGGGA
FGFR2-R	5'-TGCCAACAGTCCCTCATCAT
ACTB-F	5'-TCATTCCAAATATGAGATGCGTTGT
ACTB-R	5'-GCTATCACCTCCCCTGTGTG
GJA1-F	5'-CAAAATCGAATGGGGCAGGC
GJA1-R	5'-GCTGGTCCACAATGGCTAGT
PANX2-F	5'-CGTTCCACGTCCGCTCAC
PANX2-R	5'-GGCGATGAGGATAGCGTGTT
PANX3-F	5'-AAGTTCGTAGCTGTGGGCTC
PANX3-R	5'-CCTGCCGGATGCTGAAGTTA
LRRC8D-F	5'-CCTCCGGCCTCAGCATAAG
LRRC8D-R	5'-GCGACAGGAGACTTCACTCC
CALHM1-F	5'-TTTCTGCTTGGCCTGGTCAT
CALHM1-R	5'-AACATGTAGCGCAACACAGC
LRRC8B-F	5'-AGTCGCGCAGAATCCTCAG
LRRC8B-R	5'-ACTATTACCTGTGGGCCATCC
LRRC8E-F	5'-TTTTACACTTGCATGGCAGCAT
LRRC8E-R	5'-TGCAGCCAAAGACCCCAATC
LRRC8A-F	5'-AGAAACCAGGAGTTTCCGCCTC
LRRC8A-R	5'-AAGAAATGGCAAGGAGAGAGCC
LRRC8C-F	5'-ACAAGCCATGAGCAGCGAC
LRRC8C-R	5'-CGACTGGAGAAGGTGCTAGG
SLCO2A1-F	5'-CTGTATTTGGACCGGCTTTCG

SLCO2A1-R	5'-CCAATCCATCGGGGGTCAC
SIRT1-F	5'-GCAGATTAGTAGGCGGCTTGA
SIRT1-R	5'-TGGCATGTCCCACTATCACTG
SIRT2-F	5'-GGCAGTTCAAGCCAACCATC
SIRT2-R	5'-CCACCAAGTCCTCCTGTTCC
PPARGC1A-F	5'-TGATTGGCAGGGGCAGATTT
PPARGC1A-R	5'-CGAAGTGCTTGTTCAGCTCG
TFAM-F	5'-ACCGAGGTGGTTTTCATCTGT
TFAM-R	5'-CAACGCTGGGCAATTCTTCT
NDUFS6-F	5'-TCTTGGCCACCCAAAAGTGT
NDUFS6-R	5'-CGGAAATGCTCACAGGATGC
NDUFB4-F	5'-GGATTTGGGCCCCTCATCTT
NDUFB4-R	5'-CTGCAGCTGGTCCCTAGAAA
SDHA-F	5'-ACTGTTGCAGCACAGCTAGA
SDHA-R	5'-GCCCTTTCCAAACTTGAGGC
UQCRFS1-F	5'-ACCCAGTTCGTTTCCAGCAT
UQCRFS1-R	5'-CAGGGGTTTGCCTCTCCATT
ATP5L2-F	5'-GCCATTCGGGATGATGGACT
ATP5L2-R	5'-CAGCTCAACCGTGGTGTAGT

Supplementary Figure S1

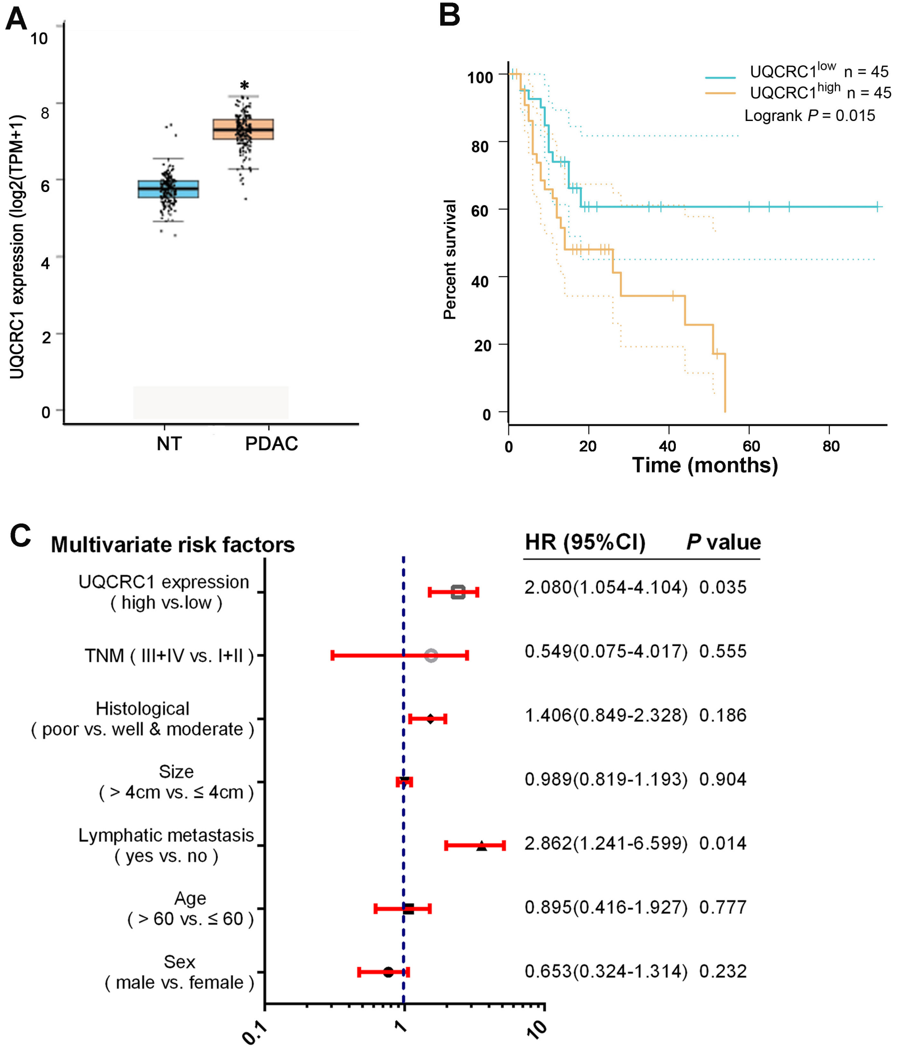

Figure S1. UQCRC1 is upregulated in PDAC and correlates with the poor prognosis of the disease in the TCGA cohort.

(A) Expression of *UQCRC1* was upregulated in PDAC patients from the TCGA compared with pancreatic tissues from the GTEx database (TCGA, n = 179; GTEx, n = 171, TPM: transcription per million). **(B)** Kaplan-Meier analysis of DFS according to the *UQCRC1* mRNA levels in 90 PDAC patients (DFS, disease-free survival). **(C)** Multivariate Cox regression analysis of the patients with PDAC in TCGA (n = 169) revealed that *UQCRC1* was an independent predictor of OS. $^{*}P < 0.05$.

Supplementary Figure S2

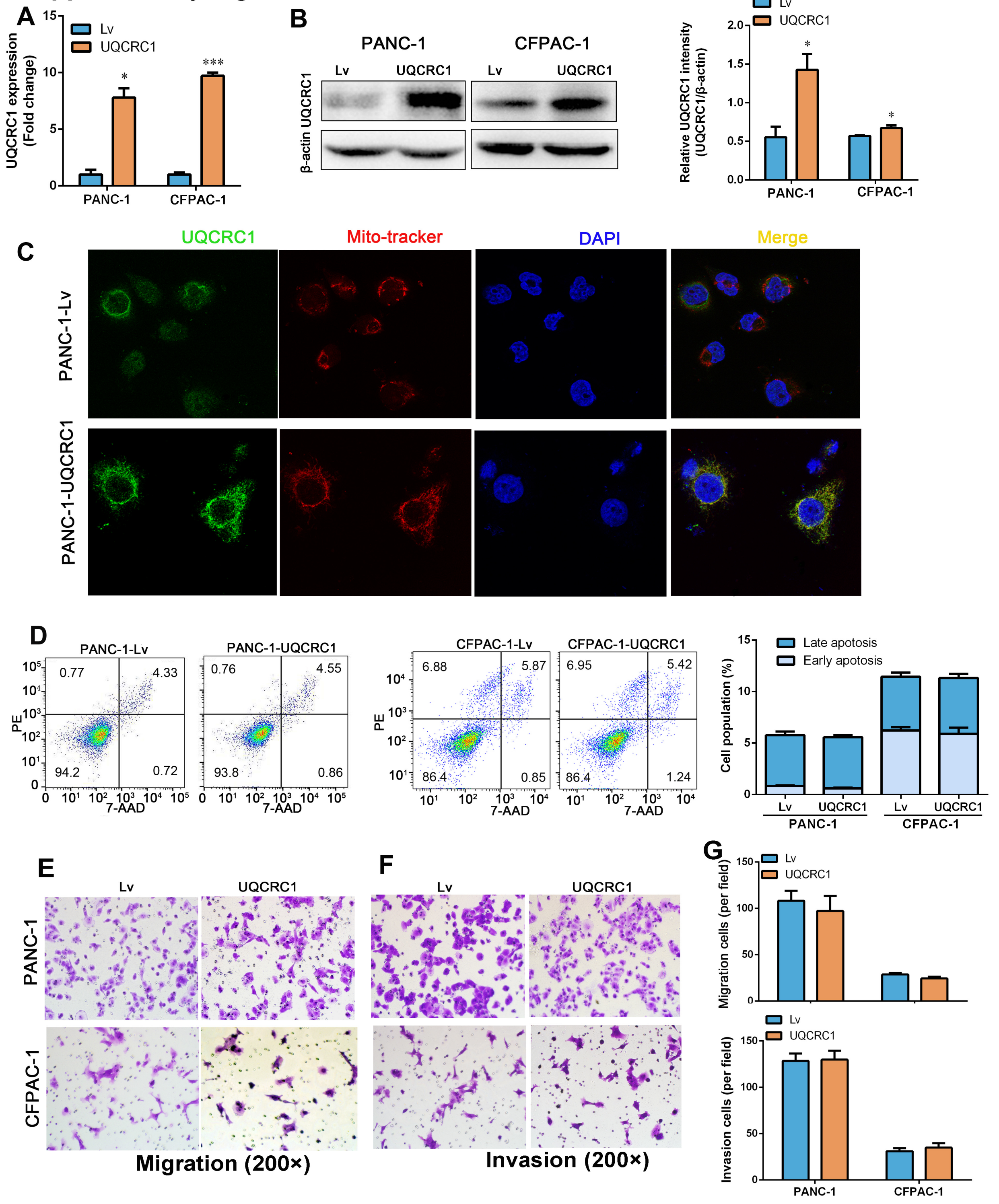

Figure S2. Overexpression of UQCRC1 has no effect on the apoptosis, migration and invasion of PDAC cells

(A) Relative mRNA and **(B)** protein levels of UQCRC1 in PANC-1 and CFPAC-1 cells infected with lentivirus carrying the *UQCRC1* gene. **(C)** Confocal microscopy indicated that ectopically expressed UQCRC1 (green) was colocalized with mitochondria (red) in PANC-1 cells. **(D)** Proapoptotic activities of UQCRC1-overexpressing PANC-1 and CFPAC-1 cells and control cells as determined by the Annexin V-PE/7-AAD assay. **(E-G)** Migration and invasion abilities of UQCRC1-overexpressing PANC-1 and CFPAC-1 cells as determined by Transwell assay (n = 3). *$P < 0.05$; ***$P < 0.001$.

Supplementary Figure S3

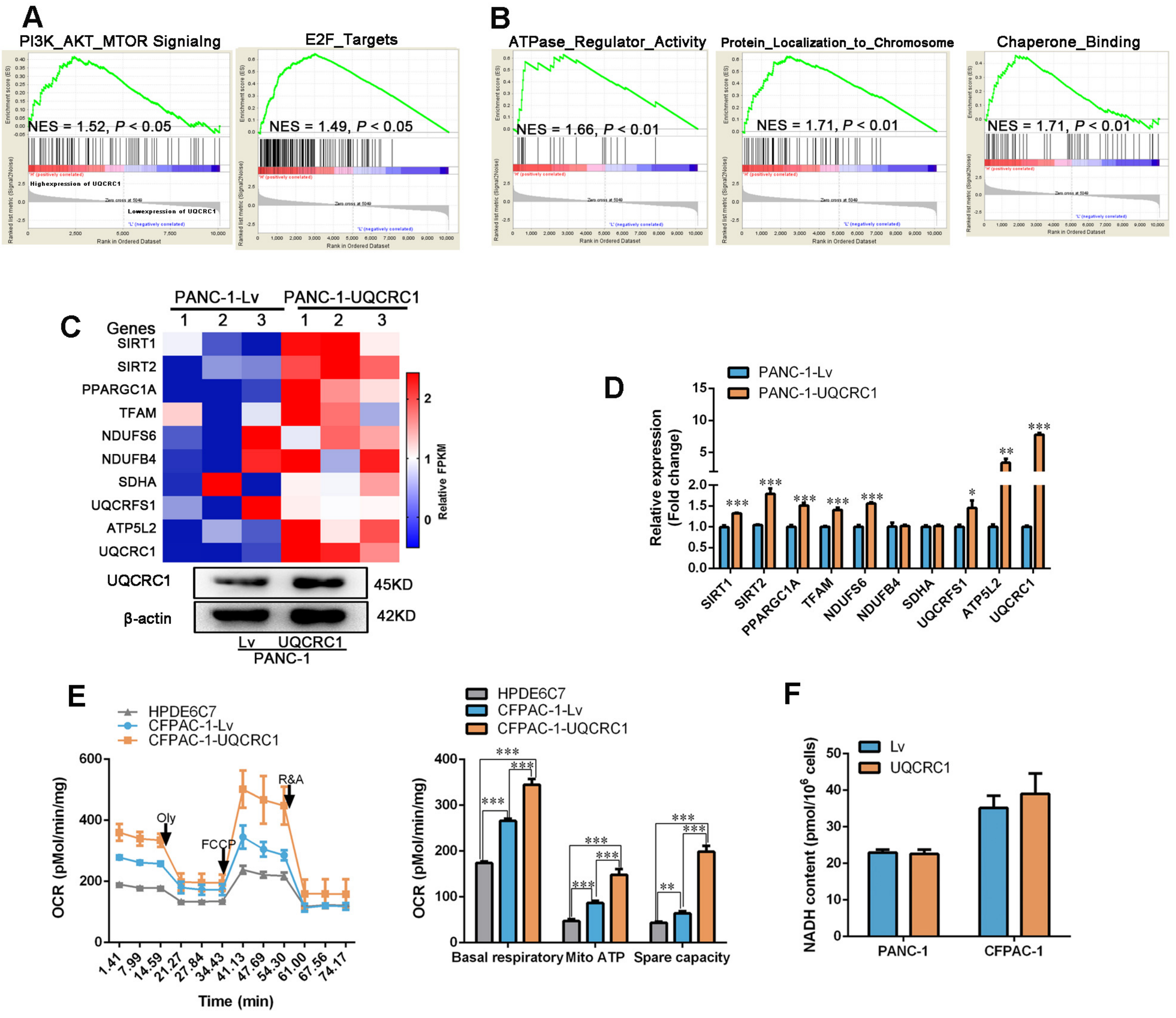

Figure S3. Overexpression of UQCRC1 enhances the OXPHOS of PDAC cells

(A) GSEA of KEGG pathway and **(B)** GO functional enrichment with RNA-Seq data (FPKM ≥ 1, a total of 10113 genes) from PANC-1-UQCRC1 and control cells. **(C)** A heat map of the genes encoding mitochondrial complexes and OXPHOS regulators in PANC-1-UQCRC1 and control cells based on the RNA-Seq profiles (n = 3, adjust $P < 0.05$). Additionally, UQCRC1 expression in PANC-1-UQCRC1 and control cells was confirmed by Western blotting. **(D)** The above genes encoding mitochondrial complexes and OXPHOS regulators in PANC-1-UQCRC1 and control cells were confirmed by qPCR (n = 3). **(E).** Increased OCRs in UQCRC1-overexpressing CFPAC-1 cells as measured by the XFe96 extracellular analyzer (n = 3; Oly, oligomycin, 1 μM; FCCP, carbonyl cyanide 4-(trifluoromethoxy) phenylhydrazone, 1 μM; R&A, rotenone and antimycin A, 1 μM; Mito, mitochondria). **(F)** NADH content in UQCRC1-overexpressing PANC-1 and CFPAC-1 cells and the control cells as determined by the NADH/NAD^+ Quantification Kit. *$P < 0.05$; **$P < 0.01$; ***$P < 0.001$.

Supplementary Figure S4

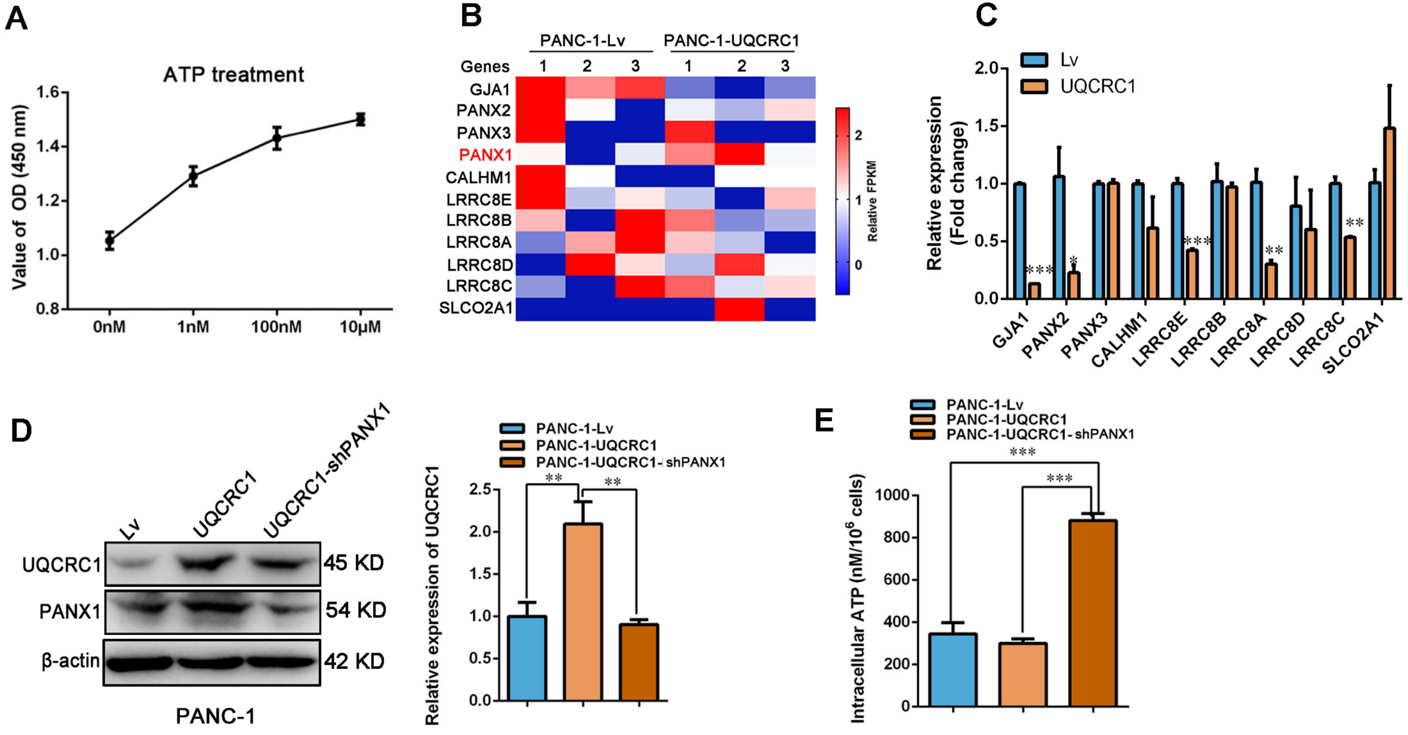

Figure S4. ATP promotes PANC-1 cell growth and downregulation of PANX1 blocks intracellular ATP release of PANC-1-UQCRC1 cells.

(A) ATP treatment from 1 nM to 10 μM for 24 h stimulated PANC-1 cell growth in a dose-dependent manner as reflected by the CCK8 assay (n = 4). **(B)** The heat map of ATP-permeable channel genes from RNA-Seq profiles. **(C)** The expression of ATP-permeable channel genes was confirmed by qPCR. **(D)** Western blot results confirmed the successful knockdown of PANX1 by shRNA in PANC-1-UQCRC1 cells. **(E)** Results of the intracellular ATP analysis of PANC-1-Lv, PANC-1-UQCRC1 and PANC-1-UQCRC1-shPANX1 cells. *P < 0.05; **P < 0.01; ***P < 0.001.

Supplementary Figure S5

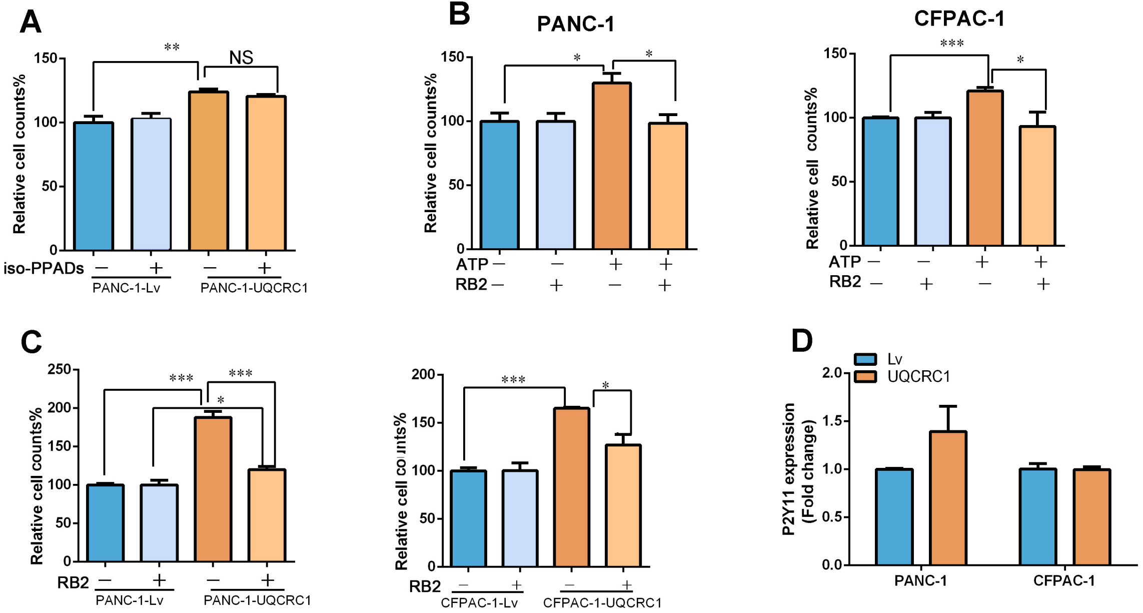

Figure S5. Identification of the membrane receptor by which eATP promotes cell proliferation

(A) Relative cell growth of UQCRC1-overexpressing PANC-1 and CFPAC-1 cells after treatment with 100 μM P2X inhibitor iso-PPADS for 48 h, as determined by the CCK8 assay (NS, nonsense, n = 4). **(B)** Relative cell growth of PANC-1 and CFPAC-1 cells after treatment with or without 10 nM ATP and/or 50 μM P2Y inhibitor RB2 for 48 h as determined by the CCK8 assay (n = 4). **(C)** Relative cell growth enhanced by UQCRC1 overexpression in PANC-1 and CFPAC-1 cells was abolished by treatment with 50 μM RB2 for 48 h, as detected by the CCK8 assay (n = 4). **(D)** Relative mRNA levels of *P2Y11* in UQCRC1-overexpressing PANC-1 and CFPAC-1 cells (n = 3). *$P < 0.05$; **$P < 0.01$; ***$P < 0.001$.

Supplementary Figure S6

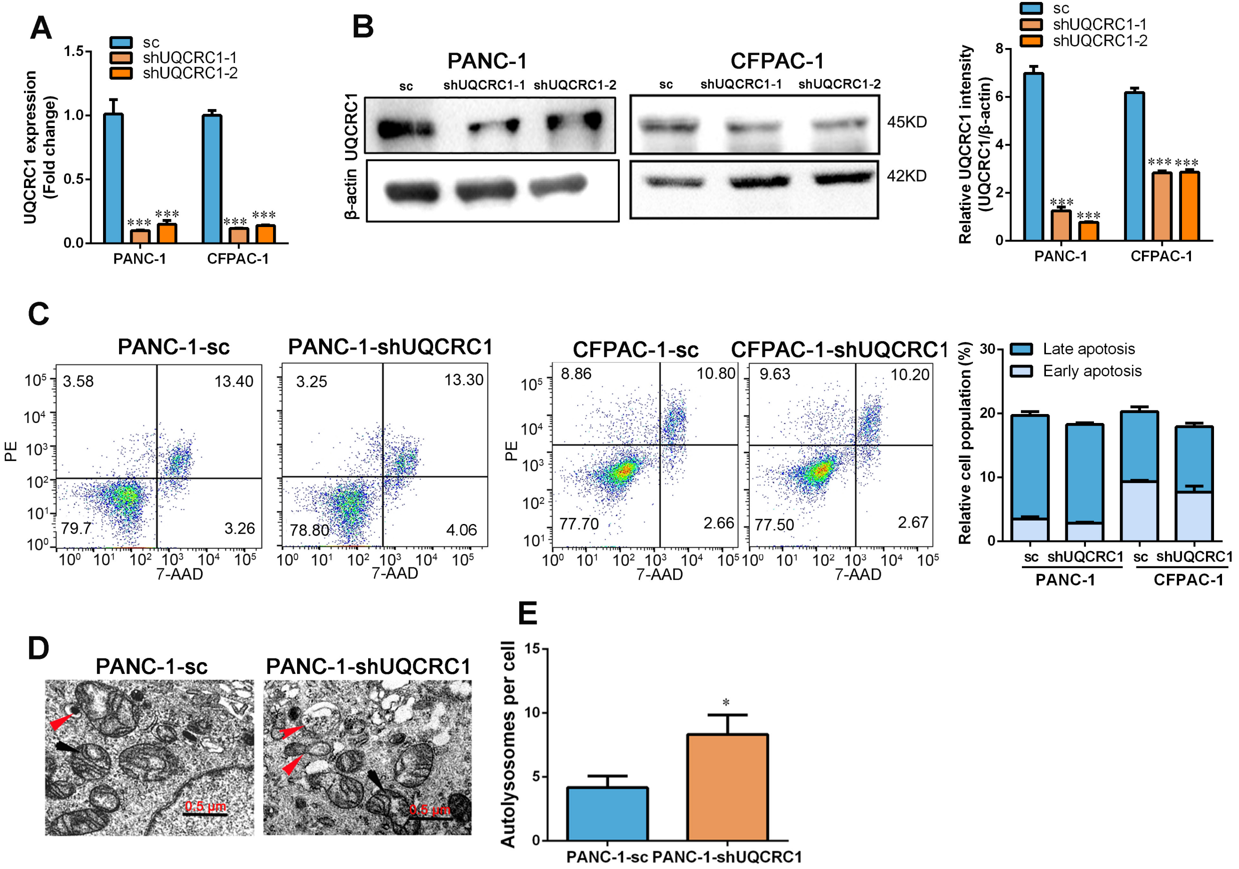

Figure S6. The effect of UQCRC1 knockdown on the apoptosis and autophagy of PDAC cells

(A) Relative mRNA and protein levels **(B)** of UQCRC1 in PDAC cells infected with shUQCRC1 or sc lentivirus (n = 3, sc: scramble). **(C)** Apoptosis remained unchanged in PANC-1 and CFPAC-1 cells with UQCRC1 knockdown, as determined by Annexin V-PE/7-AAD assay. **(D)** TEM analysis of mitochondria (black arrow) and autolysosomes (red arrow) affected by UQCRC1 downregulation in PANC-1 cells (n = 6). **(E)** Quantification of TEM data as the number of autolysosomes in the PANC-1-shUQCRC1 and control cells. *$P < 0.05$; **$P < 0.01$; ***$P < 0.001$.
